# Photochemistry and spectroscopy of small hydrated magnesium clusters Mg^+^(H_2_O)*n*, *n* = 1–5

**DOI:** 10.1063/1.5037401

**Published:** 2018-07-28

**Authors:** Milan Ončák, Thomas Taxer, Erik Barwa, Christian van der Linde, Martin K. Beyer

**Affiliations:** Institut für Ionenphysik und Angewandte Physik, Universität Innsbruck, Technikerstraße 25, 6020 Innsbruck, Austria

## Abstract

Hydrated singly charged magnesium ions Mg^+^(H_2_O)*_n_*, *n* ≤ 5, in the gas phase are ideal model systems to study photochemical hydrogen evolution since atomic hydrogen is formed over a wide range of wavelengths, with a strong cluster size dependence. Mass selected clusters are stored in the cell of an Fourier transform ion cyclotron resonance mass spectrometer at a temperature of 130 K for several seconds, which allows thermal equilibration via blackbody radiation. Tunable laser light is used for photodissociation. Strong transitions to D_1–3_ states (correlating with the 3*s*-3*p_x,y,z_* transitions of Mg^+^) are observed for all cluster sizes, as well as a second absorption band at 4–5 eV for *n* = 3-5. Due to the lifted degeneracy of the 3*p_x,y,z_* energy levels of Mg^+^, the absorptions are broad and red shifted with increasing coordination number of the Mg^+^ center, from 4.5 eV for *n* = 1 to 1.8 eV for *n* = 5. In all cases, H atom formation is the dominant photochemical reaction channel. Quantum chemical calculations using the full range of methods for excited state calculations reproduce the experimental spectra and explain all observed features. In particular, they show that H atom formation occurs in excited states, where the potential energy surface becomes repulsive along the O⋅⋅⋅H coordinate at relatively small distances. The loss of H_2_O, although thermochemically favorable, is a minor channel because, at least for the clusters *n* = 1-3, the conical intersection through which the system could relax to the electronic ground state is too high in energy. In some absorption bands, sequential absorption of multiple photons is required for photodissociation. For *n* = 1, these multiphoton spectra can be modeled on the basis of quantum chemical calculations.

## I Introduction

Hydrated magnesium ions represent an interesting system to understand the mechanisms of hydrogen production via catalysis on metal centers^1–7^ as well as corrosion effects.^8^ At the same time, they play a role in processes in the Earth’s and other planets’ upper atmosphere where magnesium is present due to the influx of interplanetary particles.^9,10^ Hydrated metal ions M^+^(H_2_O)*_n_* are well-defined moieties to study the transition of various properties from a metal atom solvated by a single water molecule to bulk behavior.^11–14^ There has been a long history of studies on microhydrated metal ions in the gas phase over the last decades. Especially, the hydrated magnesium ion has attracted considerable attention over the years^15^ and is certainly among the best studied of these systems.^16^

First *ab initio* calculations to optimize structures and determine binding energies, electronic transition energies, and vibrational frequencies of Mg^+^(H_2_O) were done by Bauschlicher, Jr. in the early 1990s.^17^ Shortly afterwards, Duncan and co-workers measured vibrationally resolved electronic and partially resolved rotational structures in the photodissociation spectrum of Mg^+^(H_2_O).^18,19^ The results confirmed the *C*_2*v*_ structure of the complex.^19^ Fuke and co-workers generated hydrated singly charged magnesium ions with up to 20 water molecules and found a dominance of Mg^+^(H_2_O)*_n_* for *n* ≤ 5 and *n* ≥ 15, whereas MgOH^+^(H_2_O)*_n-1_* species being almost exclusively present for *n* = 6-14.^20^ Photodissociation spectra for Mg^+^(H_2_O)*_n_*, *n* = 1-5, showed transitions correlating with 3*s*-3*p* excitations in Mg^+^ and provided relative cross sections and fragment branching ratios.^20,21^ Two different dissociation processes were observed: water evaporation and an intra-cluster reaction forming MgOH^+^(H_2_O)_*n*-*m*_ (m < n) and atomic hydrogen.^20,22^ A two photon process in the dissociation of Mg^+^(H_2_O)_2_ was discussed as well: The water evaporation fragments were thereby produced by only a single photon, whereas for the creation of magnesium hydroxide a switching between a single photon process and a two photon process was observed for energies lower than 3.60 eV.^20,21^ Cluster structures have been evaluated by Inokuchi *et al*. via infrared photodissociation spectroscopy on Mg^+^(H_2_O)*_n_* (*n* = 1-4).^23,24^

Iwata and co-workers investigated the structures of Mg^+^(H_2_O)*_n_* and MgOH^+^(H_2_O)_*n*-1_ (*n* = 1-6) using Møller-Plesset (MP2) perturbation theory and Multireference Configuration Interaction (MRCI) calculations to analyze these results.^25,26^ They deduced that the hydration number of the most stable structures is 3 and that the second solvation shell forms at *n* > 3;^25^ this was however questioned in subsequent studies we discuss below. The bands in the experimental spectra were assigned to an *s*-*p* transition, and, for *n* > 2, the presence of different isomers in the spectra was suggested.^26^ For *n* > 6, they proclaimed a negative energy for the hydrogen elimination process, explaining the product switching to MgOH^+^(H_2_O)_*n*-1_ observed in the experiments and for *n* > 14, Mg^2+^(H_2_O)*_n_*^−^ was presented as a candidate to explain the observed re-switching from MgOH^+^(H_2_O)_*n*-1_ to Mg^+^(H_2_O)*_n_* in the ion formation above this threshold.^25^ Plowright *et al*. revisited Mg^+^X and Mg^+^XY systems and presented structures and vibrational frequencies for Mg^+^(H_2_O)_1,2_.^9^ Dunbar and Petrie simulated the formation of Mg^+^(H_2_O) by radiative association and found it to be inefficient even at T = 10 K.^27^

Berg *et al*.^28,29^ produced Mg^+^(H_2_O)*_n_* up to *n* = 80 and measured black body infrared radiative dissociation (BIRD) rates up to *n* = 41, as well as intracluster charge transfer (CT) processes and chemical reactions. The existence of a solvent separated ion pair of Mg^2+^ and a hydrated electron for n > 17 was proclaimed by Berg *et al*.^29^ In the early 2000s, Reinhard and Niedner-Schatteburg investigated the electronic structure of Mg^+^(H_2_O)*_n_* with up to 20 water molecules.^30,31^ For *n* ≤ 5, a quasi-valence state exists; for 6 ≤ *n* < 17, a contact ion pair state exists; and for n ≥ 17, a solvent separated ion pair is formed.^30^ The existence of a hydrated electron and a Mg di-cation was proclaimed for *n* ≥ 8.^30^ Siu and Liu explained the minimum cluster size for the hydrogen loss reaction based on *ab initio* molecular dynamics calculations and investigated the influence of the coordination number on the process.^32^ They also explained the switch off for the hydrogen loss process for larger clusters due to the barrier increase when the solvated electron moves beyond the third solvation shell.^15^

Our group has a long history in studying the reactivity of hydrated metal ions,^13,16,33–47^ using Fourier transform ion cyclotron resonance mass spectrometry (FT-ICR-MS) which is well suited for this task as it allows highest mass resolution coupled with long storage times to allow multiple collisions with reactant gases. In the present study, we couple the mass spectrometer with a tunable optical parametric oscillator (OPO)/amplifier system to conduct photodissociation experiments. Experimental results for Mg^+^(H_2_O)*_n_*, *n* = 1-5, include photodissociation cross sections and product branching ratios. The results are compared with earlier experiments. Theoretical calculations are used to model the spectra and to explain the observed reactions on excited state potential energy surfaces.

## II Experimental and Theoretical Methods

The experimental setup has been described in detail earlier.^48–51^ The experiments were performed on a Fourier Transform Ion Cyclotron Resonance Mass Spectrometer (FT-ICR-MS), equipped with a 4.7 T superconducting magnet. The setup contains a laser vaporization ion source, using the 2^nd^ harmonic of a Nd:YAG laser to generate Mg^+^ ions via vaporization of a rotating target disk, consisting of isotopically enriched ^24^Mg (99.9%). Hydrated magnesium ions Mg^+^(H_2_O)*_n_* are formed via supersonic expansion into a high vacuum in a helium gas pulse seeded with water vapor, at a backing pressure of 20 bars. For storage and detection of the ions, a liquid nitrogen cooled ICR cell (T ~ 130 ± 20 K) was used to minimize the influence of blackbody infrared radiative dissociation (BIRD).^38,52–61^ Ions of a specific mass to charge ratio were isolated via resonant ejection of unwanted ions. The Mg^+^(H_2_O)*_n_* clusters were irradiated for typically 1 s by the beam of a tunable wavelength, pulsed ultra violet/visible/near-infrared (UV/VIS/NIR) laser system (Nd:YAG pumped OPO system EKSPLA NT342 B-20-SH-SFG). Typical pulse energies are shown in the supplementary material. Details on the laser setup are available elsewhere.^62^ The photon flux inside the cell was on the order of 0.1-1 mJ cm^−2^, with the strong wavelength dependence typical for OPO systems. Mass spectra of fragment and parent ions were recorded immediately after irradiation. Relative photodissociation cross sections were calculated from the parent and fragment intensities and the laser power using Lambert-Beer’s law, taking into account the contribution of BIRD to product formation (for details see the supplementary material).

The electronic ground state structures of the investigated ions were optimized with Møller-Plesset (MP2) Perturbation theory and recalculated at the Coupled Cluster with Single and Double and perturbative Triple excitations [CCSD(T)] level. The excited states were calculated using Time-Dependent Density Functional Theory (TDDFT), Equation of Motion Coupled Clusters Singles and Doubles (EOM-CCSD), Second-Order Approximate Coupled-Cluster (CC2),^63^ and Multireference Configuration Interaction (MRCI). The width of the spectra was modeled using the linearized reflection principle (LRP) within the harmonic approximation.^64–67^ For smaller clusters (*n* = 1-3), the standard reflection principle was used along with sampling of the ground state density by path integral molecular dynamics (PIMD).^68^ The latter was performed with a step size of 30 a.u., 17 000 steps per simulation out of which 3000 steps were taken as an equilibration period, and 10 random walkers (see the supplementary material for benchmark calculations). More than 700 points were used to construct each spectrum. To account for vibrational levels disregarded within the reflection principle, the spectrum for *n* = 1 was also treated within the Franck-Condon approximation, accounting for the Duschinsky rotation^69–71^ with a full-width at half-maximum of 270 cm^−1^. A basis set benchmark for modeling excited states is provided in the supplementary material, showing that aug-cc-pVDZ gives reasonable accuracy for the lowest excited states. The triple-zeta aug-cc-pVTZ basis set or another larger basis set (see Table S1 of the supplementary material) is needed for higher lying states, e.g., for the ninth excited state in doublet spin multiplicity (D_9_) and further states in Mg^+^(H_2_O).

For ground-state optimizations as well as TDDFT and EOM-CCSD calculations, Gaussian^72^ was used; MRCI calculations were performed in Molpro,^73^ CC2 calculations in Turbomole,^74^ PIMD calculations in the Abin program.^75^

## III Results and Discussion

### A Experimental photodissociation spectra

**Fig. 1:**
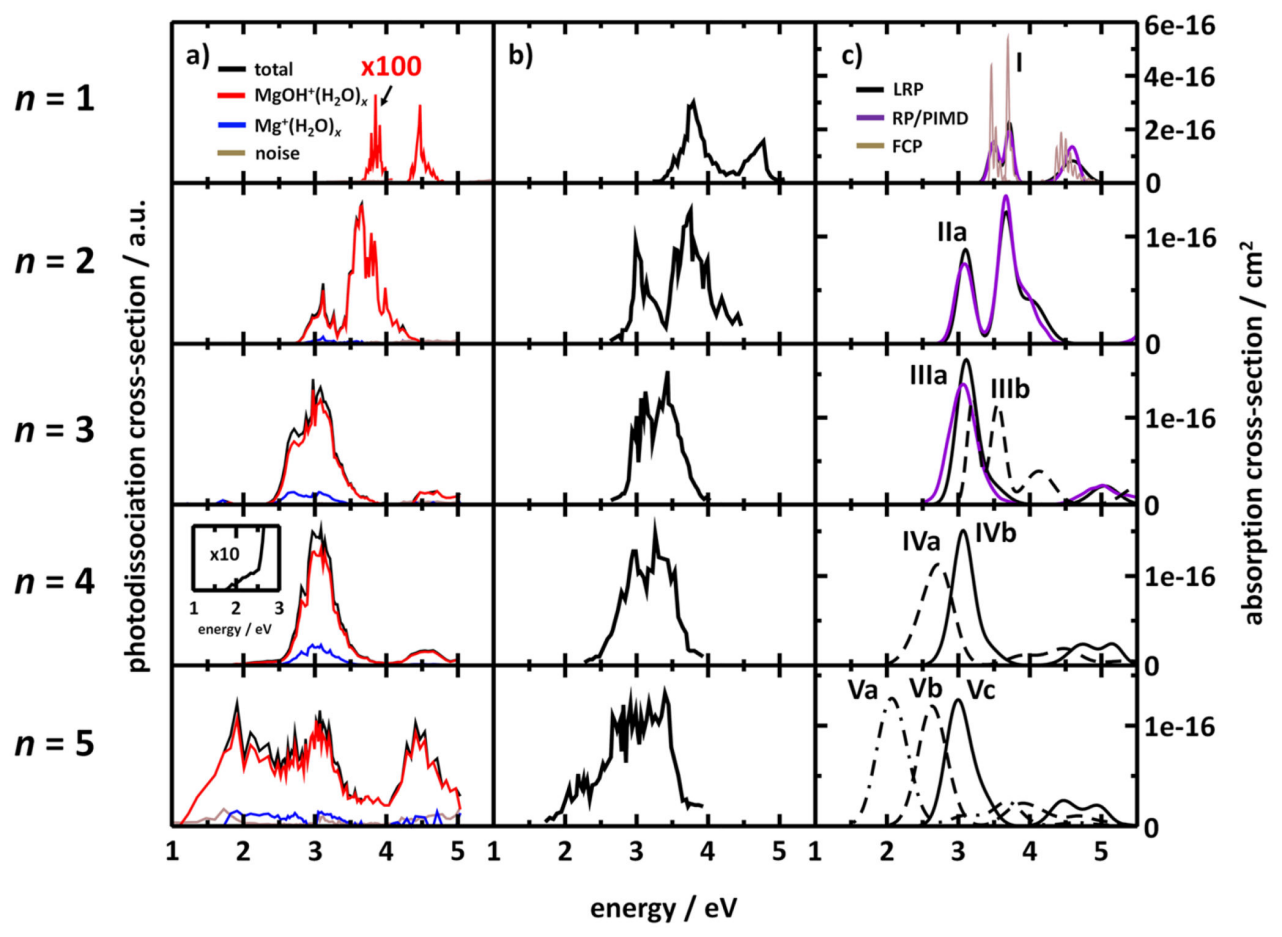
Photodissociation and photoabsorption spectra of Mg^+^(H_2_O)*_n_* clusters. (a) Photodissociation spectra measured during the present experiment. Note the relative intensity difference of about 100 for the first and second band of the Mg^+^(H_2_O) spectrum (see text for details). For *n* = 4, the spectral onset is also shown. (b) Photodissociation spectra recorded by Misaizu *et al*.^20^ (c) Modeled photoabsorption spectra of isomers shown in Fig. 2. Linearized reflection principle (LRP): Excitation energies and oscillator strengths calculated at the CC2/aug-cc-pVTZ level of theory, frequencies and forces in the excited states calculated at the CAM-B3LYP/aug-cc-pVTZ level. Reflection principle with PIMD (RP/PIMD): Sampled on the B3LYP/6-31+g* potential energy surface, with excitation energies calculated at the CC2/aug-cc-pVDZ level. Franck-Condon principle (FCP): Calculated at the EOM-CCSD/aug-cc-pVDZ level.

Measured photodissociation spectra for Mg^+^(H_2_O)*_n_*, *n* = 1–5, at 130 ± 20 K are shown in Fig. 1(a); experiments by Misaizu *et al*.^20^ are shown in Fig. 1(b) for comparison. The detected fragment ions can be grouped into two different channels. The Mg^+^(H_2_O)*_m_* (*m* < *n*) species are produced by the evaporation of one or more water molecules. The other channel, contributing most to the observed fragmentation, is the hydrogen dissociation reaction producing magnesium hydroxide MgOH^+^(H_2_O)*_m_*. The additional loss of one or more water molecules was also observed in this dissociation channel. Detailed photofragment branching ratios are provided in the supplementary material.

In the following, we denote the electronic states of Mg^+^(H_2_O)*_n_* clusters according to their irreducible representation (IR) or their number, i.e., D_1–3_ for the first absorption band and D_4+_ for the second one. However, they can also be assigned qualitatively according to the main component of the Mg orbitals, using 3*s* for the ground state and 3*s*-3*p* for excitation into the first band. We believe that this nomenclature is instructive as it connects the electronic states of hydrated Mg^+^ ions to the original states of the naked ion (see Table S3 of the supplementary material for contributing Mg^+^ orbitals and the corresponding discussion). In the second absorption band, the states correlate with the 3*d*/4*s* orbitals of Mg^+^ and interact strongly with surrounding water molecules.

For the Mg^+^(H_2_O) ion, only formation of MgOH^+^ along with the loss of a hydrogen atom is observed. The spectrum shows an intense band at 4.3–4.8 eV, with a maximum at ~4.46 eV, and a second band, two orders of magnitude less intense, at 3.8–4.0 eV, with a maximum at ~3.86 eV. The more intense band corresponds to the 3*s*-3*p_z_* transition of Mg^+^ orbitals distorted by the presence of the water molecule, whereas the less intense one corresponds to the 3*s*-3*p_x,y_* transitions, as already shown before.^17,18,76^ The Mg^+^(H_2_O) ion is also the only one for which we obtained the resolved vibrational structure, with the average splitting being 520(80) cm^−1^ and 560(160) cm^−1^ for the first and second band, respectively. For this ion, fully vibrationally resolved photodissociation spectra were measured before by Duncan and co-workers,^18,76^ who found the vibrational splitting for the first two excited states in the first absorption band to be 518 cm^−1^ for the lower energy transition and 489 cm^−1^ for the higher energy one. They also identified these frequencies as the metal-water stretching mode.

The photodissociation cross section of Mg^+^(H_2_O)_2_ consists of two bands. Here, the intensities of the bands have about the same order of magnitude. The lower energy band emerges at 2.7 eV and peaks at ~3.12 eV; the more intense band peaks at ~3.66 eV and spreads until 4.4 eV. The main dissociation channel involves again the loss of a hydrogen atom, leading to the formation of MgOH^+^(H_2_O). Fragmentation channels of H_2_O and H + H_2_O loss are observed mainly in the wavenumber region of the lower energy absorption band, at a maximum fraction of about 15%.

For Mg^+^(H_2_O)_3_, the spectrum consists of only one band at 2.3–4.1 eV, with a maximum intensity at 2.98 eV and a shoulder at 2.72 eV, red shifted by about 0.3 eV relative to the results of Misaizu *et al*.^21^ In addition to the earlier observed transitions, we document here an absorption at 4.41 eV that will be discussed in detail below. Fragmentation is again dominated by hydrogen atom loss, with MgOH^+^(H_2_O)_2_ being the most abundant product in the first band. The MgOH^+^(H_2_O) fragment ion is formed after excitation into the second band.

In the photodissociation spectrum of Mg^+^(H_2_O)_4_, the first band starts with a low intensity extended wing from 1.8 eV up to 2.5 eV where the fragmentation increases drastically (Fig. 1). The maximum is located at 3.07 eV. The upper limit of the band extends to 4.0 eV and passes smoothly into the second band that peaks at 4.6 eV. Again, hydrogen loss dominates the fragmentation for both absorption bands. For this cluster, a minor amount of BIRD fragmentation was observed. About 2%–3% of the parent ions fragmented without laser irradiation on the time scale of the experiment, with the main BIRD fragment being Mg^+^(H_2_O)_3_.

The spectrum of Mg^+^(H_2_O)_5_ consists of a broad dissociation band spanning from 1.2 eV to 4.0 eV, with a second band at 4.0–5.0 eV. The main fragment is again MgOH^+^(H_2_O)_3_, up to about 2.9 eV. BIRD fragmentation in this case is already significant, with about 9%–17% of the parent ions fragmenting without laser irradiation on the time scale of the experiment. In this case, the main BIRD fragment was MgOH^+^(H_2_O)_4_.

Comparing our measurements to the ones of the Fuke group in Fig. 1(b),^20^ we see overall good agreement, with several differences. Already for the Mg^+^(H_2_O) spectrum, apart from the vibrational resolution recorded in our measurements, there are two important features with respect to previous experiments. First, no Mg^+^ fragments were observed in our experiment, in contrast to the previously measured constant fraction of about 10% for this product.^20^ We believe that temperature or cluster preparation might play a role here. Second and more importantly, the first absorption band is about 100 times less intense in our measurements compared to the previous ones. We attribute this difference to two-photon processes as will be discussed in detail below.

Spectra of Mg^+^(H_2_O)*_n_*, *n* = 2-4, are generally consistent in both experiments, with a slightly different intensity ratio, most notably for *n* = 4. For *n* = 3, we see the abovementioned relative shift of about 0.3 eV which may be due to a different population of isomers. Similarly, for Mg^+^(H_2_O)_5_, we expect that the significant differences in spectra are caused by different populations of isomers, which in turn is caused by the different conditions during cluster preparation and the different time scales of the experiments. In the present case, the long trapping time affords thermal equilibration via the exchange of infrared photons with the environment, which is kept at ~130 K by the liquid-nitrogen cooled ICR cell. The transitions into the second band at 4–5 eV for *n* = 3–5 are reported here for the first time.

With respect to the product ions, the results are very similar for *n* = 4, 5. For *n* = 1, no water evaporation was observed in our experiments; for *n* = 2, the ratio between hydrogen loss and water evaporation is about 9:1 below 3.2 eV, whereas in the previous experiment, the ratio is about 3:2.^20^ In the same experiment, an increase in the MgOH^+^ fragment above 3.7 eV was documented, which was not seen in our experiments. For *n* = 3, the evaporation of a single water molecule is almost negligible above 2.7 eV and amounts to up to 20% below this value. Previously, it was seen to be more prominent with abundances of about 10%–40% over the whole spectrum. Another major difference for *n* = 3 is the ratio between the MgOH^+^(H_2_O)_2_ and Mg^+^(H_2_O) fragments.

### B Modeled photoabsorption spectra

**Fig. 2:**
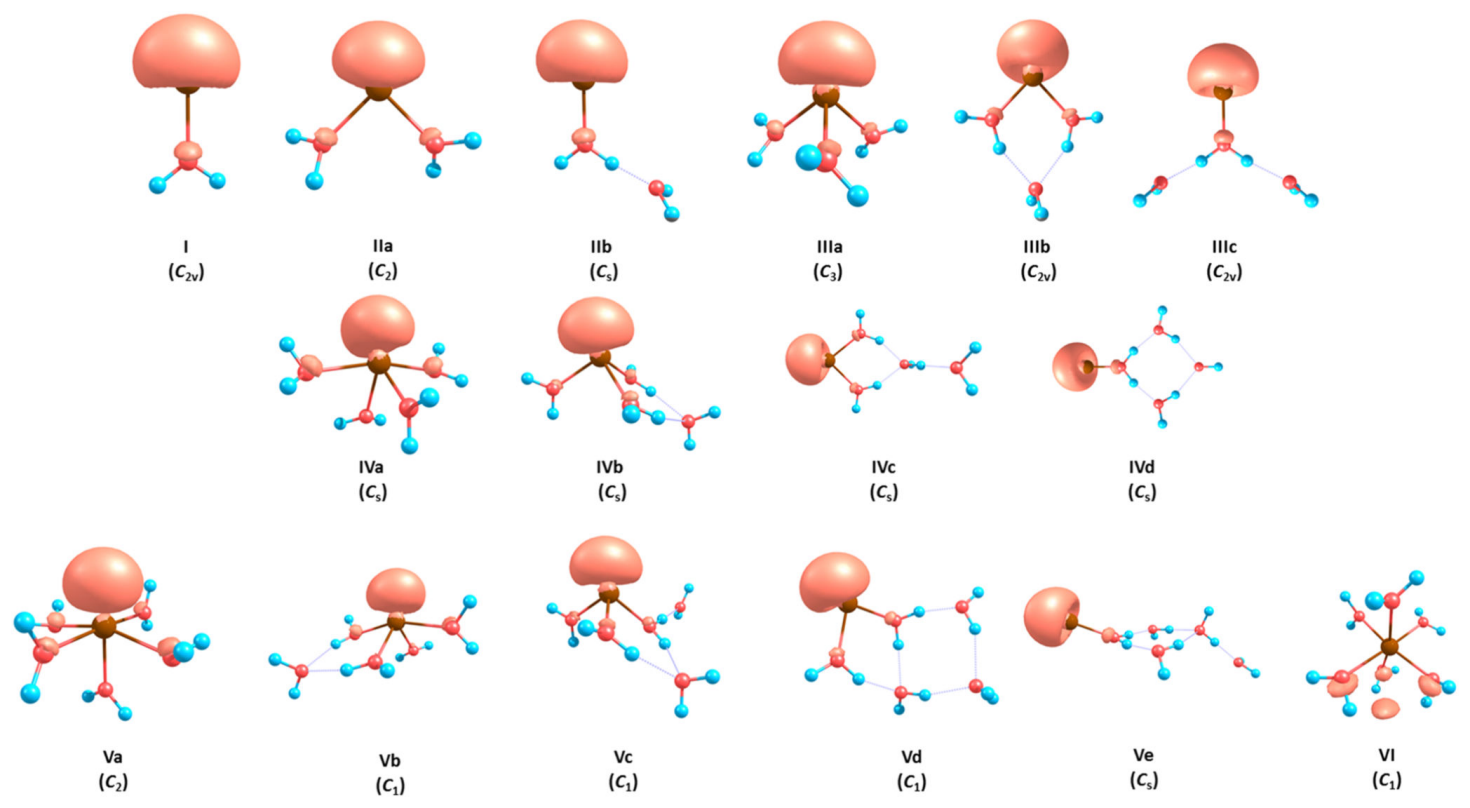
Investigated Mg^+^(H_2_O)*_n_* clusters optimized at the MP2/def2TZVP level of theory along with spin density calculated at the CCSD(T)/def2TZVP level and plotted with the isovalue of 0.006.

To model the photoabsorption spectra and photochemistry of hydrated Mg^+^, we picked several Mg^+^(H_2_O)*_n_* clusters with various bonding motifs, differing in the number of water molecules directly coordinated to the Mg^+^ ion (Fig. 2). Note that these might not necessarily represent the most stable configurations at the given level of theory. The relative stability of the clusters is summarized in Table I. As already described elsewhere,^25^ a high number of coordination bonds are preferred for *n* = 2, 3 while for *n* = 4, 5, the energy difference between three-, four-, and fivefold coordination is small, they lie within 4 kJ/mol in our calculations.

The Mg^+^(H_2_O)*_n_* system can be viewed as a Mg^+^ ion perturbed by the presence of water molecules. With continuous solvation, the 3*s* electron is eventually dissolved from Mg^+^ into water, forming Mg^2+^(H_2_O)*_n_*^−^.^25,28–30^ This dissolution is reflected in the cluster properties. Figure 2 shows the spin density of the electron. As this representation might be misleading, vertical ionization energy (VIE) and radius of gyration for the electron are also given in Table I, documenting progressive delocalization of the electron with hydration. The electron stays bound on the Mg^2+^ center, and only for *n* = 6, a considerable increase in the radius of gyration (to 2.5 Å) along with a drop in VIE (to 7.0 eV) marks the onset of significant electron delocalization. These results should be compared to the Na(H_2_O)*_n_* system that reaches a radius of gyration of more than 3.5 Å and VIE below 3.5 eV already for *n* = 4.^77^ In comparison to the average radial extent as calculated in Ref. 30, our values are systematically lower for *n* = 1–5 and comparable for *n* = 6. This difference can be traced to the fact that the radius of gyration is calculated with respect to the center of spin density, while the average radial extent was defined with respect to the Mg center. When *r*_g_ is calculated centered in Mg, a faster increase is seen, within the FWHM given in Ref. 30 (see Table S4 of the supplementary material). Quantitative differences can be explained by additional diffuse basis functions employed in the previous work.^30^

Table II summarizes the energetics of several reactions that might take place in the ground state: dissociation of H_2_O and H observed in the experiment, along with dissociation of H_2_ and OH included for comparison. Generally, all dissociation channels become more favorable with increasing hydration. The relative reaction energy for H_2_O and H dissociation channels changes with hydration, with H_2_O dissociation being preferred for a low number of water molecules and reaching about the same energy as the H dissociation channel for *n* = 4. For *n* = 5, the H dissociation channel is almost thermoneutral, with Δ*E* of 0.24 eV. H_2_ and OH dissociation channels are consistently energetically more demanding with respect to H_2_O and H dissociation. For all prominent fragments seen in the experiment, the dissociation is only observed for photon energies 0.5-1.0 eV above the calculated reaction energies for the hydrogen loss process. For water dissociation, the difference is even larger.

The data in Table II also confirm that photodissociation takes place on the excited electronic state potential energy surface, i.e., without funneling back to the electronic ground state in the vicinity of the Franck-Condon point. If this was the case, the most accessible dissociation channel would be water dissociation for *n* = 1–3, with still significant contributions for *n* = 4. The experimental data in Fig. 1, however, show that H_2_O dissociation is only a minor channel.

The character of excitation was analyzed using several *ab initio* methods. The properties of the lowest electronic transitions for *n* = 0–3 are summarized in Table III. For the Mg^+^ ion, the unpaired electron is located in the 3*s* orbital. The first three excited states correspond to excitation into the 3*p*, 4*s*, and 3*d* orbital. The 3*s*-3*p* transition has a considerable oscillator strength of about 0.3, while the other two transitions are symmetry forbidden. However, it should be noted that the 3*p*-3*d* and 3*p*-4*s* transitions are allowed, with average oscillator strengths of 0.20 and 0.15, respectively, at the MRCI(1,13)/aug-cc-pVTZ level.

For complexes of Mg^+^ with water, the respective Mg atomic orbitals are perturbed but retain their character (see Table S3 of the supplementary material). The 3*s-*3*p* transition remains bright, with oscillator strengths of about 0.3. The 3*s*-3*d*/4*s* Mg^+^ transitions correlate with electronic states in the second absorption band and become allowed since the water ligands break the symmetry. At the same time, it can be seen that excitation energies move to lower values with increasing number of water molecules, as seen in the experiment and already described by other authors.^20^

With respect to the *ab initio* methods presented in Table III, it is clear that CC2 can well reproduce both EOM-CCSD and MRCI results and will be used in the following as the method of choice for excited state calculations in the minimum geometry. TD-CAM-B3LYP, on the other hand, provides reasonably accurate results only for first few transitions (see also the supplementary material for basis set considerations).

To compare the calculated spectra with experimental data, the spectral shape was simulated employing the reflection principle both in its linearized modification and with the ground state density calculated using the PIMD method and the Franck-Condon principle [Fig. 1(c)]. Generally, both position and width of the measured spectra are well reproduced, with several exceptions that will be dealt with further. As already described before,^25^ the position of the first excitation band depends considerably on the Mg coordination number, with further water molecules in the second solvation shell playing a rather minor role. From the methodological point of view, it is apparent that the spectra produced using PIMD to sample the ground state for *n* = 1-3 are similar to the ones modeled using the linearized reflection principle; thus, it is justified to use the computationally cheaper linearized reflection principle also for the clusters with *n* = 4, 5.

For Mg^+^(H_2_O) of the *C*_2_*_v_* symmetry (lying in the *yz* plane), we see two bands, with the first band composed of 3*s*-3*p_y_*,3*p_x_* transitions, the second of 3*s*-3*p_z_* (Table S3 of the supplementary material). As discussed above, our experiment shows the former transitions to be about 100 times lower in intensity compared to the latter. The calculated photoabsorption spectrum, on the other hand, predicts two bands of comparable intensity. Here, the photodynamics of the system has to be considered (see below). While the simulated Franck-Condon spectra cannot reproduce exactly the shape of the measured spectra, the calculated vibrational progression of about 500 and 400 cm^−1^ for D_1_ and D_2_ states is in good agreement with the experimentally measured one.^18,76^

For Mg^+^(H_2_O)_2_, on the other hand, the agreement with the experiment is reached not only for position and width of the spectra, but also for the relative intensity of the absorption bands. In the case of our experiments, the intensity of the first peak is slightly lower compared to the second one. A possible involvement of two-photon processes (involving excitation from the first to the second absorption band) is analyzed below.

The Mg^+^(H_2_O)_3_ ion represents an interesting case. The experimental spectra predict a band composed of D_1–3_ states with two peaks separated by about 0.5 eV, while theory predicts two absorptions, doubly degenerate *E* and singly degenerate *A*, with the separation of about 0.3 eV (Table III). These two peaks are then smeared into one band without any apparent structure. To analyze the influence of broadening due to dynamic effects, we modeled the spectrum using the PIMD sampling [Fig. 1(c)]. However, although the resulting spectrum is slightly broader, the structure remains unchanged. Again, we can suggest that two-photon processes take place here. With respect to the spectrum measured by Fuke *et al*., its two-peak structure can be explained by accounting for isomer **IIIb** in which Mg^+^ is coordinated by only two water molecules and lies about 15 kJ/mol above triply coordinated **IIIa** (see Table I). The second absorption band of Mg^+^(H_2_O)_3_ starting at about 4.5 eV is well reproduced by the calculations with respect to both position and relative intensity, most probably with contribution of 2*E* and 3*A* states (see Table III).

Finally, for Mg^+^(H_2_O)_4_ and Mg^+^(H_2_O)_5_, there are several isomers lying close in energy (Table I) and we can expect a mixture of various structures to be present in the experiment. The differences between our and previous experimental data can be explained as dependent on the temperature and conditions in the ICR cell. In the case of *n* = 4, isomer **IVb** (or another isomer with the same Mg coordination number) seems to prevail in our experiment; however, the absorption starting at 2.0 eV can be attributed to **IVa**. In the previous experiment,^20^ both isomers **IVa** and **IVb** seem to be present to about the same extent. For *n* = 5, complexes with the Mg coordination number of 3–5 (isomers **Va**, **Vb**, and **Vc**) seem to be present in both experiments, with different relative abundances. Note however that the present experiments may be, for *n* = 5, influenced by significant BIRD contributions (see the supplementary material), which have been corrected as carefully as possible. For *n* = 4, 5, the higher lying transitions are also well reproduced by the modelled spectra, with the high-energy tail explainable by a combination of several isomers with transitions in the 4-5 eV region.

### C Photodissociation modeling

**Fig. 3:**
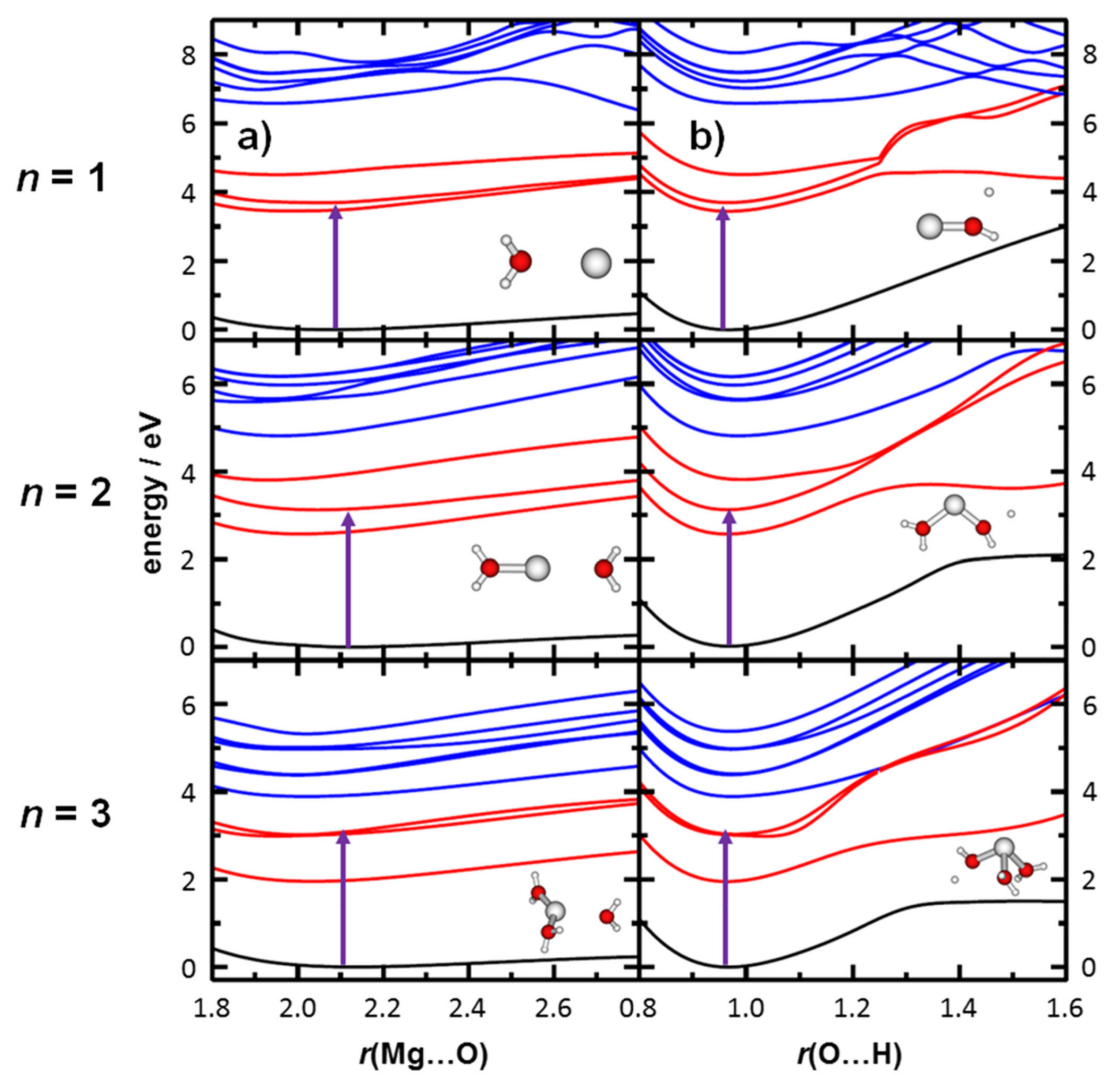
Relaxed potential energy scans for Mg^+^(H_2_O)*_n_*, *n* = 1-3, along (a) Mg-O and (b) O-H dissociation coordinate. Calculated at the EOM-CCSD/aug-cc-pVDZ level of theory, see the supplementary material for the benchmark with respect to the MRCI/aug-cc-pVDZ method. The structure was optimized in the D_0_ (black lines), D_1_ (red lines), and D_4_ (blue lines) state. The structure for the pre-dissociated ions optimized in the D_1_ state and D_0_-D_1_ excitation energy (violet arrow) in the structure optimized at the MP2/def2TZVP level are shown.

To understand the photodissociation dynamics, we calculated relaxed potential energy surface scans for the two most important dissociation coordinates found in the experiment, i.e., H-dissociation and H_2_O-dissociation, for Mg^+^(H_2_O)*_n_*, *n* = 1-3, see Fig. 3. We focus on dissociation in the ground state D_0_, in the D_1_ state as the lowest state of the first excitation band, and in the D_4_ state as the lowest state of the second excitation band.

For Mg^+^(H_2_O), only H-dissociation is observed in the experiment. Accordingly, the potential energy curves along the Mg⋅⋅⋅O coordinate are purely attractive both in the ground and in the first excitation band (Fig. 3). Only when the system is excited into D_4_ or higher states, it might dissociate with a barrier of about 7.3 eV with respect to the Franck-Condon point. A hydrogen atom, on the other hand, might pre-dissociate already within the D_1_-D_3_ band, with a barrier of about 4.5 eV. In the second band, again a barrier of about 7.3 eV is found.

As already mentioned above, the experimentally recorded hydrogen loss documents that the dissociation takes place on the excited state potential energy surface, as H_2_O dissociation would be expected when funneling back to D_0_ due to its low barrier. Moreover, potential energy surface scans show that the H-dissociation channel might not take place after excitation into D_1_ or D_2_ (at 3.4-3.7 eV, see Table III) due to the high pre-dissociation barrier. Thus, we might expect that two photons are needed in the experiment (this was also observed experimentally for the first absorption band in the analogical Ca^+^(H_2_O) system).^78^ In the D_3_ state, on the other hand, there is enough energy to surpass the barrier.

Interestingly, a similar situation arises for Mg^+^(H_2_O)_2_. Additional complexity is however added by the linearization of the ion when excited into the D_1_ and D_4_ states. The curve for water dissociation is purely attractive within the investigated region. A hydrogen atom might pre-dissociate again in both the D_1_ state with a barrier of 3.8 eV and the D_4_ state with a barrier of 6.7 eV. Again, the excitation energy into D_1_ or D_2_ states does not provide enough energy to dissociate the H atom, and we might expect a two-photon process to take place. Interestingly, the first band in the Mg^+^(H_2_O)_2_ spectrum was also previously measured to be of two-photon nature.^20^ Alternatively, the ion might collect enough energy through repeated excitation after fluorescence from the linear D_1_ minimum due to the differences of ground and excited state structure. The D_0_/D_1_ gap in the D_1_ minimum is 2.2 eV (at the EOM-CCSD/aug-cc-pVDZ level of theory), which corresponds to the energy carried away by a fluorescence photon. Back in the D_0_ minimum, the system has gained about 0.9 eV kinetic energy. Together with some initial thermal excitation, this may provide the 1.1 eV needed for water dissociation (Table II). Fluorescence into the electronic ground state would thus explain the traces of water dissociation observed in our experiment. Additional thermal excitation may also open the pathway for non-radiative decay to the electronic ground state through conical intersections, which may explain the difference with respect to previous experiments.

For Mg^+^(H_2_O)_3_, we see the same situation, with no barrier on the dissociative curves for both H_2_O and H dissociation. After excitation in D_1_ or D_4_ states, the ion becomes planar, with an energy gain of about 1 eV. Dissociation of a water molecule is hindered by a purely attractive potential in all investigated electronic states, and this channel is suppressed in the experiment. Most probably, the H-dissociation observed in the experiment takes place at least partially through two-photon processes as the expected dissociation barrier is higher than the excitation energy into the D_1_ state, i.e., 3 eV (Fig. 3).

According to our calculations, the water dissociation channel (seen in our experiment for *n* > 1) can be explained in two ways. First, water dissociation coordinate may be activated within two-photon processes after reaching D_4_ or higher states where enough energy is available due to structural relaxation. Alternatively, it might be reached through fluorescence back to the ground state or due to opening non-radiative channels to D_0_. Due to significant structural changes in the D_1_ state for *n* > 1, the ion has enough energy to dissociate a water molecule after switching back into the D_0_ state.

### D Simulation of the mixed one- and two-photon photodissociation spectrum for Mg^+^(H_2_O)

**Fig. 4:**
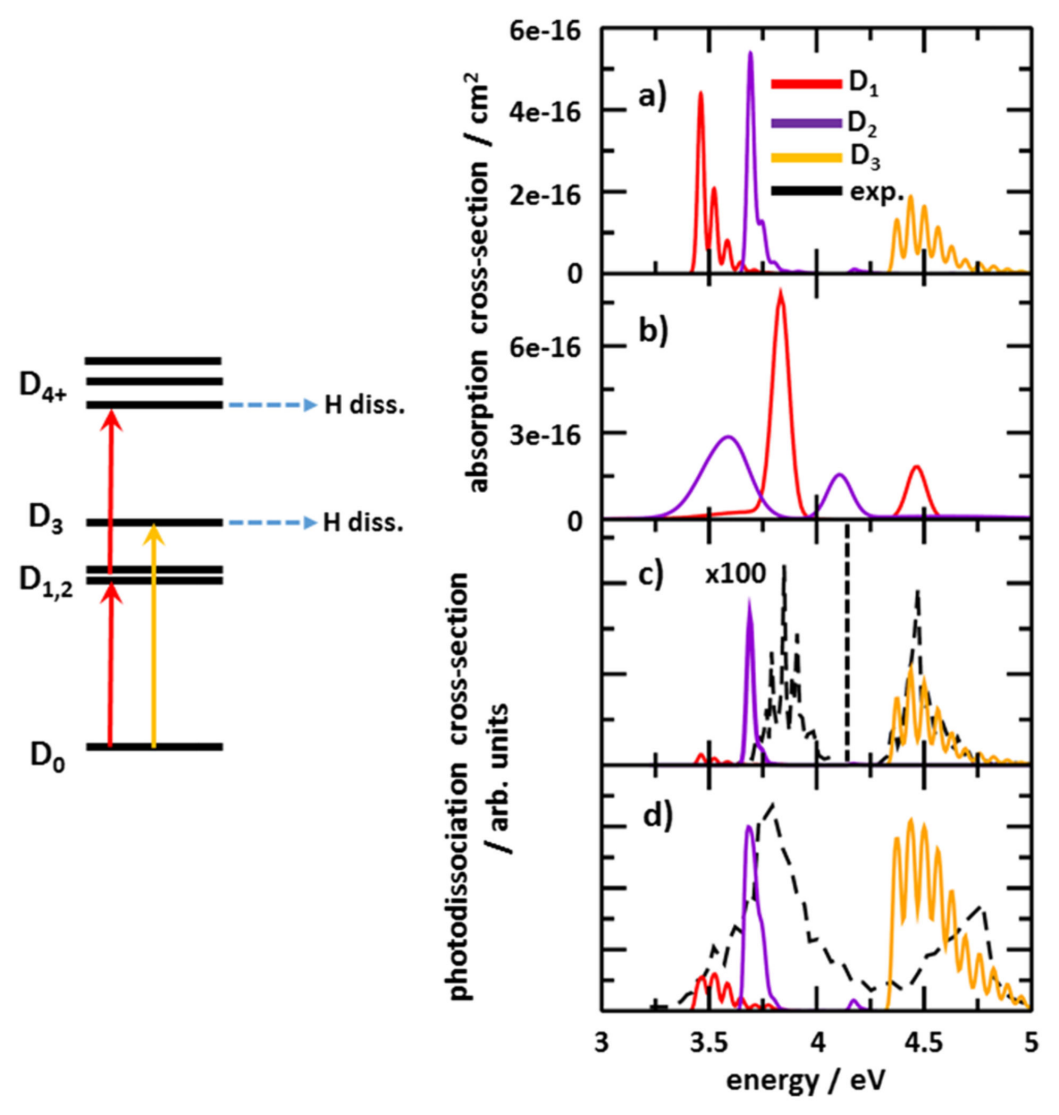
Modeled photodissociation spectra. Left-hand side: Scheme used to model the photodissociation spectra. Right-hand side: (a) Absorption spectra for the first photon calculated using the Franck-Condon approximation, decomposed into D_1_-D_3_ states. Calculated at the EOM-CCSD/aug-cc-pVDZ level of theory. (b) Absorption spectra for the second photon calculated using the linearized reflection principle approximation for excitation starting from D_1_ and D_2_ minima. Calculated at the EOM-CCSD/aug-cc-pVDZ level of theory, with the EOM-CCSD/aug-cc-pVTZ used for excitation energies and MRCI(1,14)/aug-cc-pVTZ for transition dipole moments between excited states. (c) Modeled photodissociation spectra for low laser flux in comparison with the present experimental results. (d) Modeled photodissociation spectra for high laser flux in comparison with the data of Misaizu *et al*.^20^

The analysis of potential energy surface scans has shown that hydrogen dissociation in Mg^+^(H_2_O) might take place after absorption of two photons (initial excitation in D_1_, D_2_ states) or one photon (initial excitation in D_3_), see scheme in Fig. 4. Using this scheme, we modeled the photodissociation spectrum by direct simulations of the ion interaction with a laser pulse. For this purpose, the absorption spectrum for the first photon was modeled using the Franck-Condon approximation [Fig. 4(a)]. The absorption of the second photon in the D_1_ and D_2_ states was calculated using the linearized reflection principle in the respective excited state minima [Fig. 4(b)].

To model the two-photon photodissociation spectra shown in Figs. 4(c) and 4(d), we simulated the direct interaction of the laser pulse with the ion based on the calculated photoabsorption spectra. We decomposed the laser pulse into finite time steps of 10^−12^ s and approximated it with a Gaussian curve. We picked two different values of laser flux, 100 and 0.4 J/m^2^/pulse (further denoted as high and low flux, respectively) that turned out to reproduce the experimental spectra, see below. We modeled the total probability of promoting the system into the given state by integrating along the interaction of the laser pulse with the system in time. For the states that do not dissociate directly (D_1_, D_2_), the second photon can be absorbed within the remaining duration of the laser pulse or fluorescence might take place, with the rate given by the respective Einstein coefficient. The molecule was assumed to dissociate once the D_3_ or higher states were reached.

Within the low laser flux limit, we simulate the situation when the D_1_/D_2_ states are rather little populated. With this laser flux choice, we can reproduce the relative intensity of the D_1_/D_2_ and D_3_ absorption bands measured in our experiment [Fig. 4(c)]. While the position and width of the one-photon D_3_ band can be well reproduced by our calculation, the D_1_/D_2_ band is off by about 0.2 eV and its structure is smeared. Under the approximations used, we consider both spectra to be in good agreement.

For the high laser flux, we obtain considerably populated D_1_/D_2_ states, with further excitation into higher states occurring within a *pseudo* one-photon process. Here, we are able to reproduce the relative intensity of both bands as recorded in the experiment of Misaizu *et al*. [Fig. 4(d)].^20^ With respect to the peak width and structure, the D_1_/D_2_ band seems to be better reproduced by our calculations, again with a shift of about 0.2 eV with respect to the maximum. Note that this band is also broader compared to the one with the scaled photon flux in Fig. 4(c), in agreement with the experimental trend. The position and structure of the D_3_ band could be reproduced only semi-quantitatively. Here, the neglect of temperature influence represents an important source of error.

By comparing our experimental data with the theoretical model and using the measurements of the Duncan group that recorded the onset of the D_2_ transition in Mg^+^(H_2_O) at ~3.75 eV,^18^ we can conclude that the first band sampled in our measurement is probably exclusively the D_2_ state, with D_1_ absorption lying lower in energy and expected to be of only very limited intensity as shown in our photodissociation spectra modeling [Fig. 4(c)]. In the previous experiment of the Fuke group,^20^ on the other hand, we expect both D_1_ and D_2_ states to be smeared into the first absorption band.

We have shown that different relative peak intensities in both experiments can be reproduced by considering low and high photon flux interacting with the ions. This provides evidence for two-photon absorption in D_1_/D_2_ states and one-photon for D_3_. Based on this modeling and the potential energy scans presented in Fig. 3, we expect two-photon processes to play an important role also for *n* = 2, 3. Here, the photodissociation modeling is complicated by significant structural changes after excitation.

## IV Conclusions

Relative photodissociation cross sections were measured for Mg^+^(H_2_O)_1-5_ clusters in the range of 0.6–5.0 eV. The results are overall in good agreement with the theoretical predictions, as well as earlier experiments,^20^ although the dissociation bands all seem to be shifted to the red, especially in the case of the Mg^+^(H_2_O)_3_ cluster. A second dissociation band was observed in this work for the Mg^+^(H_2_O)_3–5_ clusters. The BIRD influence was documented at cell temperatures of *T* ~ 130 ± 20 K, at the rate of 2%–3% and 9%–17% for Mg^+^(H_2_O)_4_ and Mg^+^(H_2_O)_5_, respectively. It was found that for the Mg^+^(H_2_O)_1–3_ clusters, only a single isomer was present, whereas several isomers contribute to the dissociation spectrum of Mg^+^(H_2_O)_4,5_. For all investigated cluster sizes, hydrogen dissociation producing MgOH^+^(H_2_O)*_m_* was the main observed dissociation channel.

By analysis of potential energy curves and photodissociation spectra modeling, we have shown that two photons are needed for hydrogen dissociation in D_1_ and D_2_ states of Mg^+^(H_2_O). We argue that, for a high photon flux, absorption of two photons proceeds in a *pseudo* one-photon regime. This behavior might also be expected for *n* = 2, 3 (as already indicated for Mg^+^(H_2_O)_2_ in a previous study^20^). However, simulations for these systems will be more challenging due to the large amount of energy released during structural changes after excitation.

## Supplementary Material

See supplementary material for method benchmarks, details on analysis of the experimental data, and Cartesian coordinates of calculated ions and molecules.

Supporting Information

## Figures and Tables

**Table I:** Properties of Mg^+^(H_2_O)_*n*_ clusters: relative energy *ΔE*, vertical ionization energy VIE, and gyration radius *r_g_* as evaluated from the spin density. Calculated at the CCSD(T)/def2TZVP//MP2/def2TZVP level.

Cluster	∆*E* (kJ/mol)	VIE (eV)	*r*_g_ (Å)
Mg^+^	⋅⋅⋅	14.7	1.62
I	⋅⋅⋅	12.8	1.66
IIa	0.0	11.2	1.70
IIb	30.7	12.1	1.66
IIIa	0.0	10.0	1.74
IIIb	15.0	10.7	1.70
IIIc	58.5	11.6	1.66
IVa	3.5	9.0	1.84
IVb	0.0	9.4	1.75
IVc	25.2	10.3	1.70
IVd	81.3	11.3	1.67
Va	3.5	8.0	1.98
Vb	0.0	8.5	1.83
Vc	2.0	9.0	1.76
Vd	39.4	10.0	1.70
Ve	93.3	11.1	1.67
VI	⋅⋅⋅	7.0	2.52

**Table II:** Reaction energy of various dissociation reactions of Mg^+^(H_2_O)*_n_* ions (in eV). Structures were optimized at the MP2/def2-TZVP level of theory and single-point recalculated at the CCSD(T)/def2-TZVP level. Zero-point correction is included at the MP2/def2-TZVP level.

Reaction\cluster	Mg^+^H_2_O	Mg^+^(H_2_O)_2_	Mg^+^(H_2_O)_3_	Mg^+^(H_2_O)_4_	Mg^+^(H_2_O)_5_
Mg^+^(H_2_O)_*n*_ → Mg^+^(H_2_O)_*n*-1_ + H_2_O	1.38	1.12	0.95	0.69	0.59
Mg^+^(H_2_O)_*n*_ → MgOH^+^(H_2_O)_*n*-1_ + H	2.99	1.92	1.20	0.57	0.24
Mg^+^(H_2_O)_*n*_ → MgH^+^(H_2_O)_*n*-1_ + OH	4.38	3.56	3.02	2.50	2.30
Mg^+^(H_2_O)_*n*_ → MgO^+^(H_2_O)_*n*-1_ + H_2_	4.00	2.91	2.13	1.43	1.05

**Table III:** Excitation energies (in eV) and oscillator strengths (in parentheses) using various methods with the aug-cc-pVTZ basis set, unless stated otherwise, in the structures optimized at the MP2/def2TZVP level of theory. State description with respect to the state number and irreducible representation (IR) is given. The *D*_2*h*_ symmetry group was used for Mg^+^. Only the most stable isomers were considered. See Tables S1 and S2 of the supplementary material for further details.

Ion	State	IR	EOM-CCSD	MRCI(1,*X*)^a^	CC2	TD-CAM-B3LYP
Mg^+^	D_1–3_	*B_u_*	4.28 (3.2 × 10^−1^)	4.27 (3.2 × 10^−1^)	4.32 (3.1 × 10^−1^)	4.70 (3.2 × 10^−1^)
	D_4_	*A_g_*	8.45 (0.0 × 10^+0^)	8.44 (0.0 × 10^+0^)	8.49 (0.0 × 10^+0^)	8.68 (0.0 × 10^+0^)
	D_5-9_	*A_g_, B_g_*	8.68 (0.0 × 10^+0^)	8.65 (0.0 × 10^+0^)	8.72 (0.0 × 10^+0^)	8.79 (0.0 × 10^+0^)
Mg^+^H_2_O	D_1_	1*B*_2_	3.46 (2.5 × 10^−1^)	3.45 (2.6 × 10^−1^)	3.50 (2.4 × 10^−1^)	3.77 (2.4 × 10^−1^)
	D_2_	1*B*_1_	3.68 (2.6 × 10^−1^)	3.67 (2.7 × 10^−1^)	3.72 (2.6 × 10^−1^)	3.99 (2.5 × 10^−1^)
	D_3_	2*A*_1_	4.54 (2.9 × 10^−1^)	4.57 (3.0 × 10^−1^)	4.60 (3.0 × 10^−1^)	4.63 (2.7 × 10^−1^)
	D_4_	3*A*_1_	6.63 (4.3 × 10^−2^)	6.66 (3.7 × 10^−2^)	6.71 (4.0 × 10^−2^)	6.48 (6.5 × 10^−2^)
	D_5_	2*B*_2_	7.20 (5.3 × 10^−5^)	7.24 (3.7 × 10^−4^)	7.29 (7.6 × 10^−5^)	7.01 (8.5 × 10^−4^)
	D_6_	4*A*_1_	7.24 (8.1 × 10^−3^)	7.23 (5.6 × 10^−3^)	7.28 (7.4 × 10^−3^)	7.34 (8.9 × 10^−3^)
	D_7_	1*A*_2_	7.34 (0.0 × 10^+0^)	7.30 (0.0 × 10^+0^)	7.38 (0.0 × 10^+0^)	7.39 (0.0 × 10^+0^)
	D_8_	5*A*_1_	7.35 (1.1 × 10^−3^)	7.31 (1.5 × 10^−3^)	7.40 (1.4 × 10^−3^)	7.38 (2.7 × 10^−3^)
Mg^+^(H_2_O)_2_	D_1_	1*B*	3.07 (2.2 × 10^−1^)	3.06 (2.4 × 10^−1^)	3.10 (2.2 × 10^−1^)	3.29 (2.1 × 10^−1^)
	D_2_	2*B*	3.62 (2.8 × 10^−1^)	3.65 (2.9 × 10^−1^)	3.66 (2.8 × 10^−1^)	3.71 (2.5 × 10^−1^)
	D_3_	2*A*	3.94 (1.8 × 10^−1^)	4.02 (1.9 × 10^−1^)	4.00 (1.9 × 10^−1^)	3.81 (1.6 × 10^−1^)
	D_4_	3*A*	5.65 (7.6 × 10^−2^)	5.64 (6.7 × 10^−2^)	5.70 (7.5 × 10^−2^)	5.57 (7.8 × 10^−2^)
	D_5_	3*B*	5.70 (8.8 × 10^−3^)	5.76 (6.7 × 10^−3^)	5.78 (7.0 × 10^−3^)	5.42 (2.5 × 10^−2^)
	D_6_	4*A*	6.00 (1.8 × 10^−4^)	6.01 (6.7 × 10^−5^)	6.07 (1.8 × 10^−7^)	5.82 (8.8 × 10^−3^)
	D_7_	4*B*	6.23 (2.2 × 10^−4^)	6.23 (4.3 × 10^−5^)	6.31 (3.0 × 10^−4^)	6.03 (1.3 × 10^−3^)
	D_8_	5*A*	6.24 (2.9 × 10^−2^)	6.21 (2.1 × 10^−2^)	6.28 (2.8 × 10^−2^)	6.27 (3.0 × 10^−2^)
Mg^+^(H_2_O)_3_	D_1,2_	1*E*	3.09 (2.4 × 10^−1^)^b^	3.12 (2.7 × 10^−1^)^b^	3.10 (2.4 × 10^−1^)	3.09 (2.2 × 10^−1^)
	D_3_	2*A*	3.37 (1.2 × 10^−1^)^b^	3.49 (1.3 × 10^−1^)^b^	3.41 (1.2 × 10^−1^)	3.13 (1.1 × 10^−1^)
	D_4,5_	2*E*	4.86 (8.4 × 10^−3^)^b^	4.92 (7.5 × 10^−3^)^b^	4.89 (6.3 × 10^−3^)	4.57 (2.2 × 10^−2^)
	D_6_	3*A*	5.05 (7.7 × 10^−2^)^b^	5.02 (8.7 × 10^−2^)^b^	5.06 (8.2 × 10^−2^)	4.93 (4.0 × 10^−2^)
	D_7,8_	3*E*	⋅⋅⋅	⋅⋅⋅	5.41 (5.0 × 10^−4^)	5.11 (2.3 × 10^−4^)

a (1,13), (1,14), (1,11), and (1,7) active spaces were used for Mg^+^, Mg^+^H_2_O, Mg^+^(H_2_O)_2_, and Mg^+^(H_2_O)_3_, respectively.

b Calculated with the aug-cc-pVDZ basis set.
