# Supplementary material for: Photochemistry and spectroscopy of small hydrated magnesium clusters Mg+(H2O)n, n = 1–5
Source: J Chem Phys. Author manuscript; Available in PMC 2020 Mar 16. (PMC7075709; doi:10.1063/1.5037401)
Supplement: Supporting Information [file EMS85923-supplement-Supporting_Information.pdf]

Supporting Information

Photochemistry and Spectroscopy of Small Hydrated Magnesium Clusters $Mg^+(H_2O)_n$, n = 1–5

Milan Ončák,* Thomas Taxer, Erik Barwa, Christian van der Linde, Martin K. Beyer*

Institut für Ionenphysik und Angewandte Physik, Leopold-Franzens-Universität Innsbruck, Technikerstraße 25, 6020 Innsbruck, Austria

*Corresponding authors: milan.oncak@uibk.ac.at; martin.beyer@uibk.ac.at

1. Benchmark of computational methods, electronic states character

Table S1 – Excitation energies (in eV) and oscillator strengths (in parenthesis) using the EOM-CCSD method with various basis sets, in the structures optimized at the MP2/def2TZVP level of theory. Only the most stable isomers were considered.

ion	state	def2TZVP	def2QZVPPD	aug-cc-pVDZ	aug-cc-pVTZ	aug-cc-pVQZ
$Mg(H_2O)^+$	$1B_2$	3.50 (2.5E-1)	3.47 (2.5E-1)	3.46 (2.5E-1)	3.46 (2.5E-1)	3.46 (2.47E-1)
	$1B_1$	3.72 (2.7E-1)	3.69 (2.6E-1)	3.68 (2.6E-1)	3.68 (2.6E-1)	3.68 (2.60E-1)
	$2A_1$	4.63 (3.0E-1)	4.54 (2.9E-1)	4.54 (2.9E-1)	4.54 (2.9E-1)	4.53 (2.94E-1)
	$3A_1$	7.07 (4.2E-2)	6.65 (4.3E-2)	6.63 (4.3E-2)	6.63 (4.3E-2)	6.62 (4.25E-2)
	$2B_2$	7.90 (6.8E-3)	7.27 (1.8E-4)	7.20 (5.3E-5)	7.20 (5.3E-5)	7.19 (9.8E-5)
	$4A_1$	7.66 (8.0E-3)	7.31 (8.9E-3)	7.24 (8.1E-3)	7.24 (8.1E-3)	7.22 (7.5E-3)
	$1A_2$	7.68 (0.0E+0)	7.37 (0.0E+0)	7.35 (0.0E+0)	7.34 (0.0E+0)	7.28 (0.0E+0)
	$5A_1$	7.83 (1.1E-2)	7.37 (2.4E-3)	7.34 (1.1E-3)	7.35 (1.1E-3)	7.29 (2.0E-3)
	$2B_1$	8.19 (2.2E-2)	7.80 (3.2E-3)	7.82 (3.9E-3)	7.82 (3.9E-3)	7.70 (2.9E-3)
	$3B_2$	8.63 (3.0E-2)	7.98 (2.7E-2)	7.94 (2.6E-2)	7.94 (2.6E-2)	7.87 (2.4E-2)
	$3B_1$	8.40 (6.1E-3)	8.27 (2.5E-2)	8.20 (2.4E-2)	8.20 (2.4E-2)	-
	$4B_1$	9.62 (2.1E-2)	8.38 (3.1E-4)	8.29 (1.1E-5)	8.29 (1.0E-5)	-
	$6A_1$	10.66 (2.0E-3)	8.93 (1.1E-2)	8.78 (5.2E-3)	8.78 (5.2E-3)	-
	$4B_2$	11.70 (4.5E-3)	8.90 (4.3E-3)	8.84 (5.1E-3)	8.84 (5.1E-3)	-
$Mg(H_2O)_2^+$	$1B$	3.11 (2.3E-1)	-	3.07 (2.2E-1)	3.07 (2.2E-1)	-
	$2B$	3.71 (2.9E-1)	-	3.62 (2.8E-1)	3.62 (2.8E-1)	-
	$2A$	4.16 (2.1E-1)	-	3.94 (1.8E-1)	3.94 (1.8E-1)	-
	$3A$	6.17 (7.3E-2)	-	5.65 (7.6E-2)	5.65 (7.6E-2)	-
	$3B$	6.36 (5.1E-3)	-	5.70 (8.8E-3)	5.69 (8.8E-3)	-
	$4A$	6.90 (1.7E-2)	-	6.00 (1.8E-4)	6.00 (1.8E-4)	-
	$4B$	7.28 (6.1E-3)	-	6.23 (2.2E-4)	6.23 (2.2E-4)	-
	$5A$	6.96 (4.9E-2)	-	6.24 (2.9E-2)	6.24 (2.9E-2)	-
	$6A$	7.39 (1.1E-2)	-	6.68 (3.9E-3)	6.68 (3.9E-3)	-
	$5B$	7.87 (2.8E-2)	-	6.98 (2.3E-2)	6.98 (2.3E-2)	-
$Mg(H_2O)_3^+$	$1E$	3.20 (2.6E-1)	-	3.09 (2.4E-1)	-	-
	$2A$	3.72 (1.3E-1)	-	3.37 (1.2E-1)	-	-
	$2E$	5.57 (5.0E-3)	-	4.86 (8.4E-3)	-	-
	$3A$	6.15 (2.0E-1)	-	5.05 (7.7E-2)	-	-
	$3E$	6.48 (2.2E-3)	-	-	-	-

Table S2 – Excitation energies (in eV) and oscillator strengths (in parenthesis) using various methods with the aug-cc-pVTZ basis set, unless stated otherwise, in the structures optimized at the MP2/def2TZVP level of theory. Only the most stable isomers were considered.

ion	state	EOM-CCSD	MRCI(1,*X*)[a]	CC2	TD-CAM-B3LYP
Mg^+	3*p*	4.28 (3.2E-1)	4.27 (3.2E-1)	4.32 (3.1E-1)	4.70 (3.1E-1)
	4*s*	8.45 (0.0E+0)	8.44 (0.0E+0)	8.49 (0.0E+0)	8.68 (0.0E+0)
	3*d*	8.68 (0.0E+0)	8.65 (0.0E+0)	8.72 (0.0E+0)	8.79 (0.0E+0)
$Mg(H_2O)^+$	$1B_2$	3.46 (2.5E-1)	3.45 (2.6E-1)	3.50 (2.4E-1)	3.77 (2.4E-1)
	$1B_1$	3.68 (2.6E-1)	3.67 (2.7E-1)	3.72 (2.6E-1)	3.99 (2.4E-1)
	$2A_1$	4.54 (2.9E-1)	4.57 (3.0E-1)	4.60 (3.0E-1)	4.63 (2.7E-1)
	$3A_1$	6.63 (4.3E-2)	6.66 (3.7E-2)	6.71 (4.0E-2)	6.48 (6.5E-2)
	$2B_2$	7.20 (5.3E-5)	7.24 (3.7E-4)	7.29 (7.6E-5)	7.01 (8.5E-4)
	$4A_1$	7.24 (8.1E-3)	7.23 (5.6E-3)	7.28 (7.4E-3)	7.34 (8.9E-3)
	$5A_1$	7.34 (1.1E-3)	7.31 (1.5E-3)	7.40 (1.4E-3)	7.38 (2.7E-3)
	$1A_2$	7.35 (0.0E+0)	7.30 (0.0E+0)	7.38 (0.0E+0)	7.39 (0.0E+0)
	$2B_1$	7.82 (3.9E-3)	7.77 (3.1E-3)	7.89 (3.7E-3)	7.07 (1.3E-2)
	$3B_2$	7.94 (2.6E-2)	7.92 (1.8E-2)	7.99 (2.5E-2)	7.90 (2.7E-2)
	$3B_1$	8.20 (2.4E-2)	8.20 (4.7E-3)	8.13 (2.3E-2)	7.82 (3.7E-3)
	$4B_1$	8.29 (1.1E-5)	10.08 (3.4E-3)	8.32 (2.7E-3)	8.27 (5.8E-3)
	$6A_1$	8.78 (5.2E-3)	8.71 (6.3E-3)	8.83 (5.8E-3)	8.72 (3.7E-3)
	$4B_2$	8.84 (5.1E-3)	-	8.91 (5.4E-3)	8.69 (4.3E-3)
$Mg(H_2O)_2^+$	1*B*	3.07 (2.2E-1)	3.06 (2.4E-1)	3.10 (2.2E-1)	3.29 (2.1E-1)
	2*B*	3.62 (2.8E-1)	3.65 (2.9E-1)	3.66 (2.8E-1)	3.71 (2.5E-1)
	2*A*	3.94 (1.8E-1)	4.02 (1.9E-1)	4.00 (1.9E-1)	3.81 (1.6E-1)
	3*A*	5.65 (7.6E-2)	5.64 (6.7E-2)	5.70 (7.5E-2)	5.57 (7.8E-2)
	3*B*	5.70 (8.8E-3)	5.76 (6.7E-3)	5.78 (7.0E-3)	5.42 (2.5E-2)
	4*A*	6.00 (1.8E-4)	6.01 (6.7E-5)	6.07 (1.8E-07)	5.82 (8.8E-3)
	4*B*	6.23 (2.2E-4)	6.23 (4.3E-5)	6.31 (3.0E-4)	6.03 (1.3E-3)
	5*A*	6.24 (2.9E-2)	6.21 (2.1E-2)	6.28 (2.8E-2)	6.27 (3.0E-2)
	6*A*	6.68 (3.9E-3)	6.61 (4.6E-3)	6.73 (3.6E-3)	6.61 (2.5E-3)
	5*B*	6.98 (2.3E-2)	6.92 (1.4E-2)	7.03 (2.3E-2)	6.89 (2.2E-2)
$Mg(H_2O)_3^+$	1*E*	3.09 (2.4E-1)[b]	3.12 (2.7E-1)[b]	3.10 (2.43E-1)	3.09 (2.2E-1)
	2*A*	3.37 (1.2E-1)[b]	3.49 (1.3E-1)[b]	3.41 (1.20E-1)	3.13 (1.1E-1)
	2*E*	4.86 (8.4E-3)[b]	4.92 (7.5E-3)[b]	4.89 (6.3E-3)	4.57 (2.2E-2)
	3*A*	5.05 (7.7E-2)[b]	5.02 (8.7E-2)[b]	5.06 (8.2E-2)	4.93 (4.0E-2)
	3*E*	-	-	5.41 (5.0E-4)	5.11 (2.3E-4)

a) (1,13), (1,14), (1,11), and (1,7) active spaces were used for Mg^+, $Mg(H_2O)^+$, $Mg(H_2O)_2^+$, $Mg(H_2O)_3^+$, respectively

b) calculated with the aug-cc-pVDZ basis set

Table S3 analyzes the character of the four lowest electronic states in $Mg^+(H_2O)_n$ isomers and its correlation to the states of the bare Mg^+ ion, i.e. 3*s* and 3*p*. For the smallest clusters, the assignment is clear and almost quantitative, with a 3*s* the ground state and three 3*p* electronically excited states, whose degeneracy is lifted by the lower symmetry. The *s* and $p_{y,z}$ atomic orbitals (AOs) are often the dominant contributions to the respective molecular orbital (MO). For the correlating p_z states, only a limited contribution of the Mg AOs to the total MO are observed. For larger clusters with H_2O in the second solvation shell (**IVb**, **Vb**, **Vc**), all Mg^+ *s* and *p* orbitals contribute to the respective states, making the qualitative analysis less clear. However, all excitations into the first band can be described as, at least partial, 3*s*-3*p* transitions as the 3*s* component always dominates the ground state and there is always a strong 3*p* component in the excited state.

Table S3 – Analysis of the character of the first four electronic states of various $Mg^+(H_2O)_n$ isomers, calculated at the TD-CAM-B3LYP/aug-cc-pVTZ level of theory in the structure optimized at the MP2/def2TZVP level. The character of the state was calculated by summing the square of the AO coefficients for MOs that compose the given electronic state. All MOs with contribution higher than 6% for the given state were included. “Mg contribution” shows the relative weight of Mg orbitals for the given state.

Isomer	state	character	Mg contribution / %	assignment
**I**	$1A_1$	96% *s* / 4% p_z	93	3*s*
	$1B_2$	100% p_y	95	3p_y
	$1B_1$	100% p_x	97	3p_x
	$2A_1$	2% *s* / 98% p_z	39	3p_z
**Iia**	1A	87% *s* / 13% p_z	89	3*s*
	1B	70% p_x / 30% p_y	62	3$p_{x,y}$
	1B	73% p_x / 27% p_y	63	3$p_{x,y}$
	2A	13% *s* / 87% p_z	22	3p_z
**IIIa**	1A	79% *s* / 21% p_z	85	3*s*
	1E	50% p_x / 50% p_y	26	3$p_{x,y}$
	2A	19% *s* / 81% p_z	27	3p_z
**IVa**	1A'	79% *s* / 8% p_x / 13% p_y	77	3*s*
	2A'	28% *s* / 45% p_x / 26% p_y	28	3*s*/3$p_{x,y}$
	1A''	100% p_z	12	3p_z
	3A'	18% *s* / 26% p_x / 56% p_y	26	3$p_{x,y}$
**IVb**	1A'	78% *s* / 22% p_x	83	3*s*
	2A'	49% *s* / 33% p_x / 18% p_y	19	3*s*/3$p_{x,y}$
	1A''	100% p_z	20	3p_z
	3A'	43% *s* / 28% p_x / 29% p_y	21	3*s*/3$p_{x,y}$
**Va**	1A	84% *s* / 16% p_z	70	3*s*
	1B	96% p_x / 4% p_y	15	3p_x
	2B	4% p_x / 96% p_y	14	3p_y
	2A	44% *s* / 56% p_z	39	3*s*/3p_z
**Vb**	1A	77% *s* / 2% p_x / 21% p_z	78	3*s*
	2A	71% *s* / 9% p_x / 19% p_z	32	3*s*/3*p*
	3A	48% *s* / 9% p_x / 28% p_y / 14% p_z	24	3*s*/3*p*
	4A	41% *s* / 11% p_x / 36% p_y / 13% p_z	19	3*s*/3*p*
**Vc**	1A	77% *s* / 5% p_y / 17% p_z	83	3*s*
	2A	53% *s* / 6% p_x / 10% p_y / 30% p_z	20	3*s*/3*p*
	3A	35% *s* / 29% p_x / 22% p_y / 15% p_z	16	3*s*/3*p*
	4A	32% *s* / 32% p_x / 19% p_y / 17% p_z	18	3*s*/3*p*

Table S4 – Radius of gyration (in Å) $r_{g,Mg}$ of the unpaired electron for Mg^+ and several $Mg^+(H_2O)_n$ isomers calculated with respect to the Mg center. Calculated at the CCSD(T)/def2TZVP//MP2/def2TZVP level.

system	$r_{g,Mg}$
Mg^+	1.62
**I**	1.72
**IIa**	1.84
**IIIa**	1.97
**Iva**	2.15
**Va**	2.41
**VI**	3.15

2. Calculation of the relative photodissociation cross sections

Relative intensities were calculated by determining the photodissociation cross sections σ according to Equation 1.

Equation 1: $I_0 = \sum_{i=0}^{n} I_i \, e^{-\sigma \frac{\lambda p E}{h c A} - k_{BIRD} \, t}$

Here I_0 is the parent ion's intensity and $I_{1\text{-}n}$ are the intensities of the n fragment ions. The laser wavelength is represented by λ, the number of pulses by *p* and the pulse energy by *E*. Planck's constant is *h*, *c* is the speed of light and *A* represents the irradiated area inside the cell. The constant for the BIRD rate is k_{BIRD} and *t* is the time the ions were exposed to black body radiation.

The set up for the laser irradiation is shown in Figure S1.

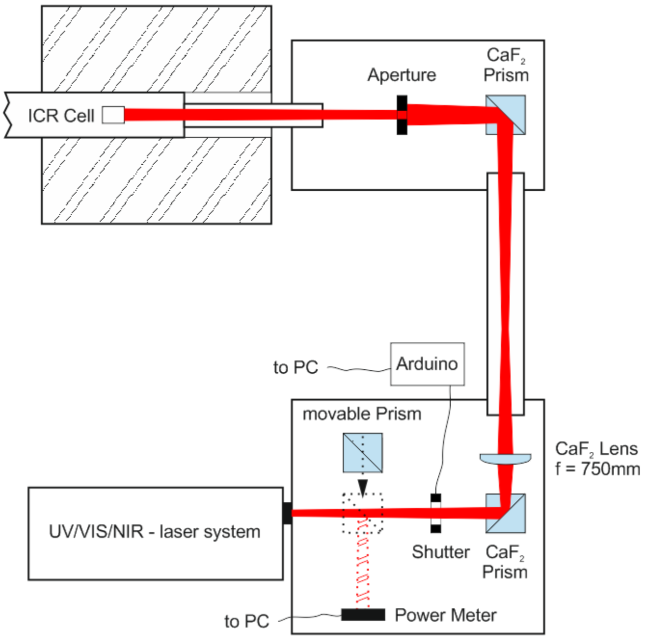

Figure S1: Optical set up for the experiments

The number of pulses *p* is controlled by an electronically controlled shutter. The pulse energies *E* are measured by a power meter. Figure S2 shows some power spectra of the laser system in the relevant wavelength range.

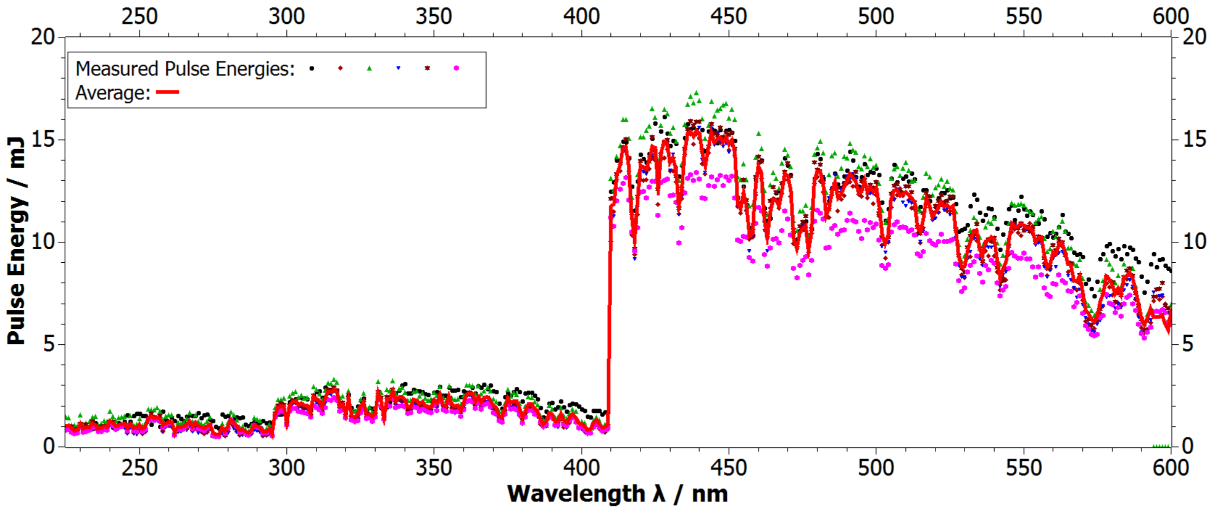

Figure S2: Power spectra of the laser system used for ion irradiation.

Measurements of the pulse energies were performed behind an aperture at a distance of 3.4m from the ICR cell and compared to measurements directly in front of the laser (Figure S3) to get an estimate of the photon flux inside the cell.

3. Detailed figures with all dissociation channels

Figures S3 – S7 show the detailed photodissociation spectra of the investigated ions, along with their branching ratios.

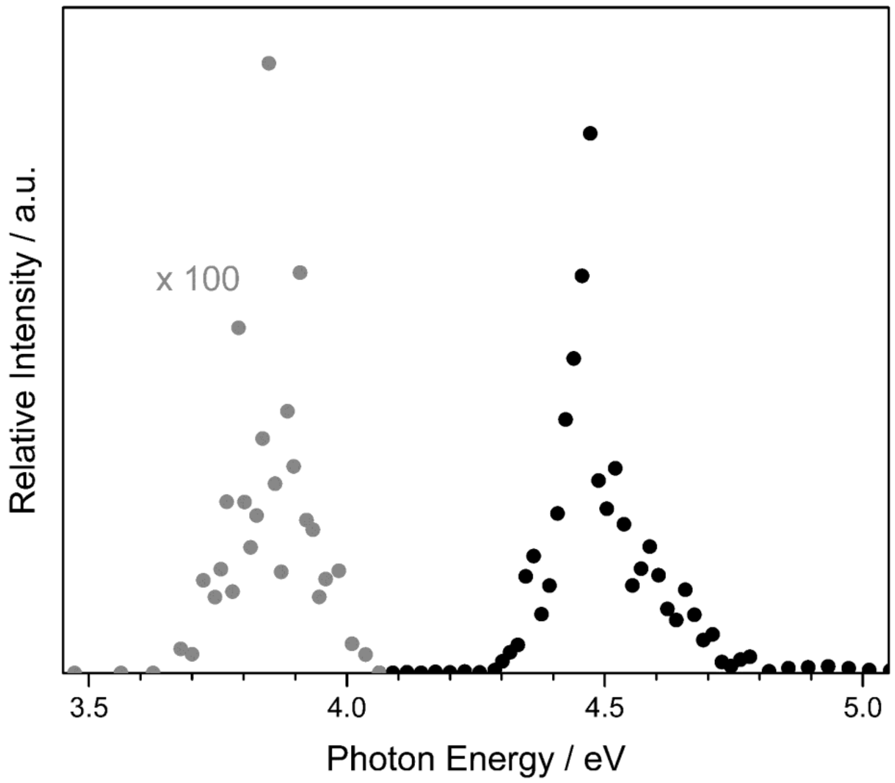

Figure S3: Photodissociation spectrum of $Mg^+(H_2O)$. The only observed fragment was $MgOH^+$.

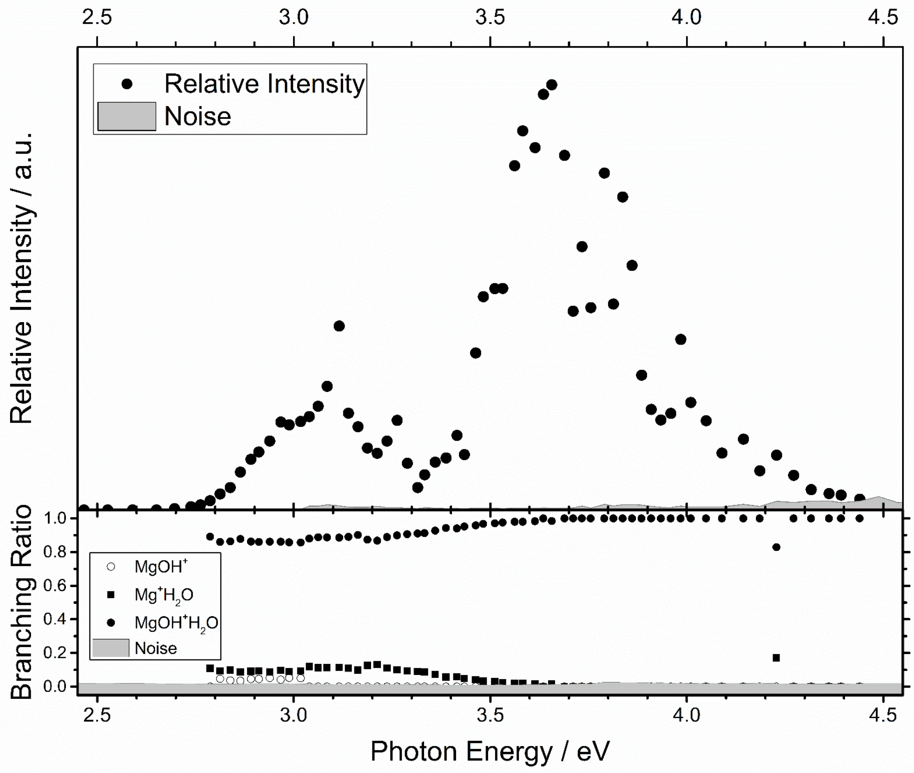

Figure S4: Photodissociation spectrum and branching ratios of $Mg^+(H_2O)_2$

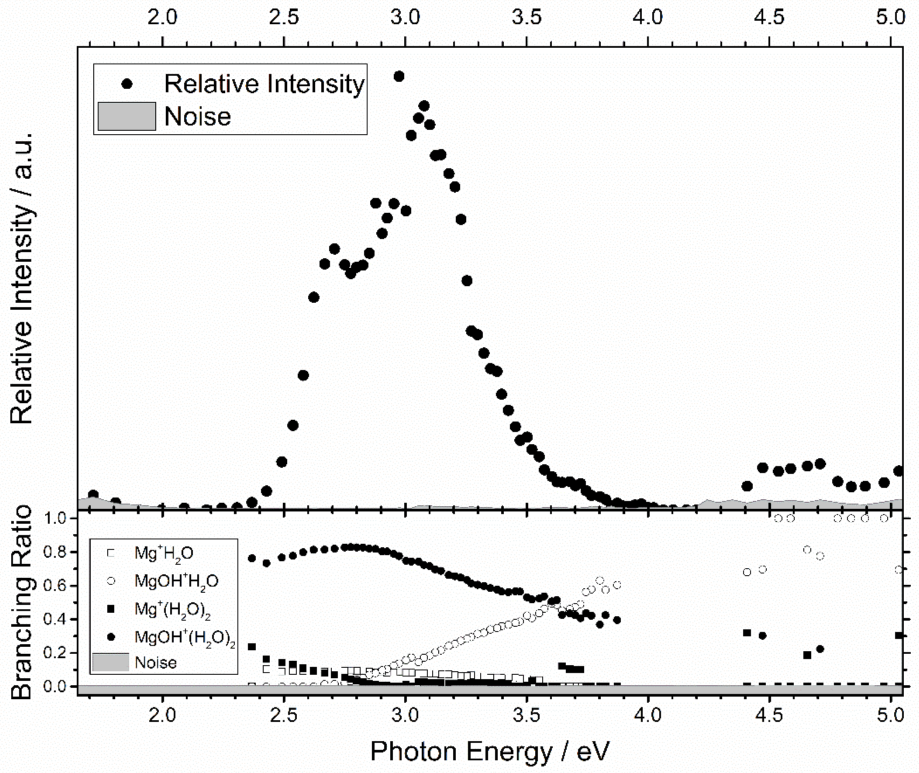

Figure S5: Photodissociation spectrum and branching ratios of $Mg^+(H_2O)_3$

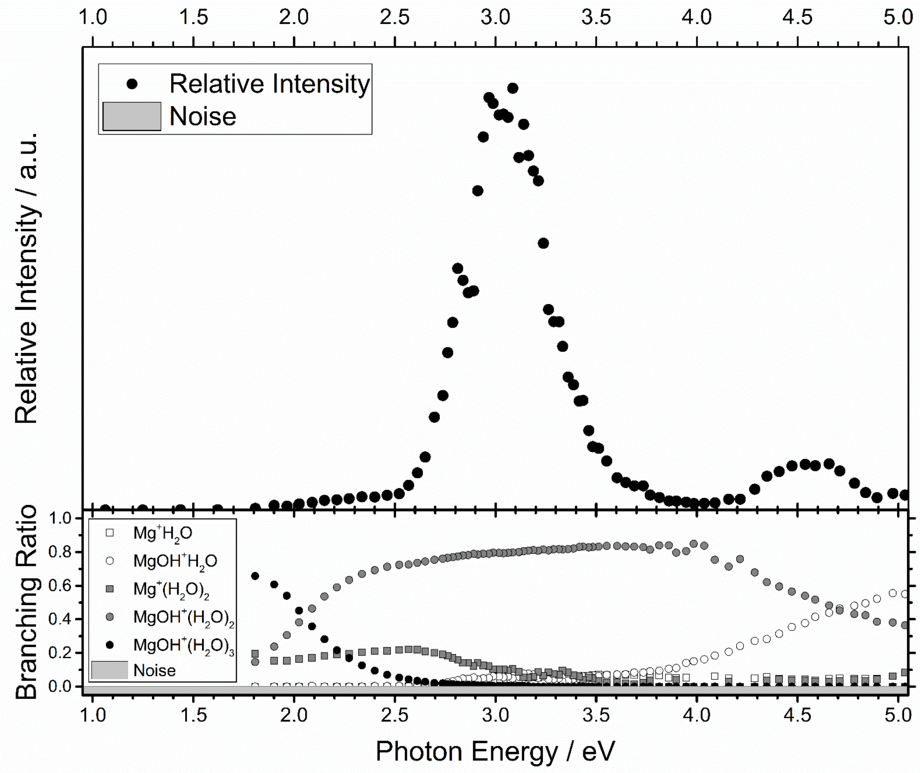

Figure S6: Photodissociation spectrum and branching ratios of $Mg^+(H_2O)_4$

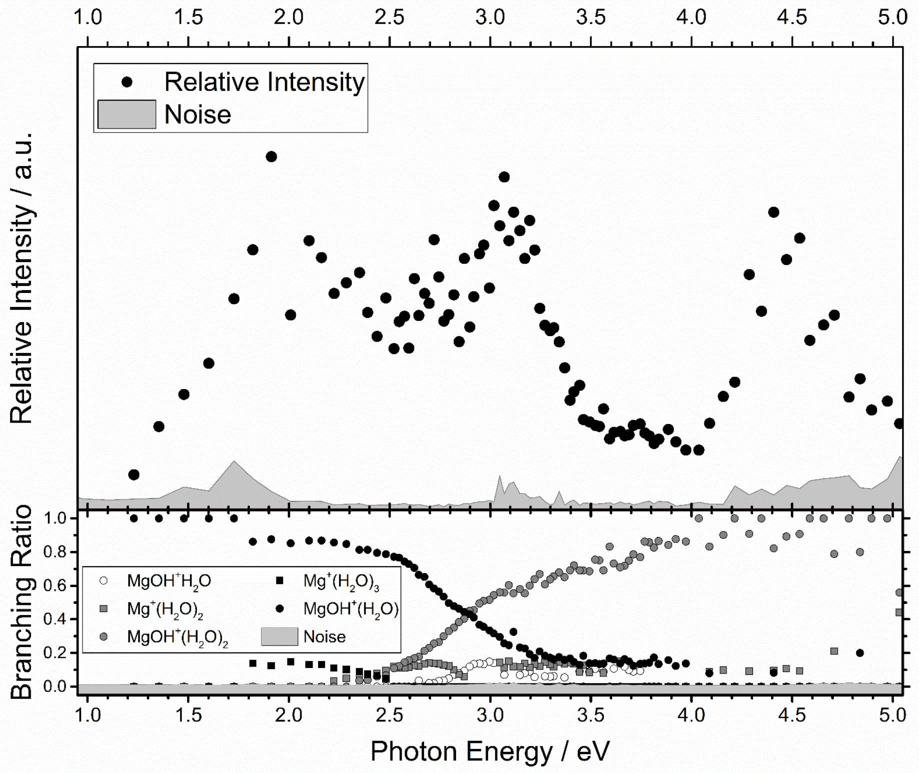

Figure S7: Photodissociation spectrum and branching ratios of $Mg^{+}(H_2O)_5$

4. Reconstruction of the single photon spectrum for $Mg(H_2O)^+$

A reconstruction of the single photon absorption spectrum for the Mg^+H_2O ion is shown in figure S8.

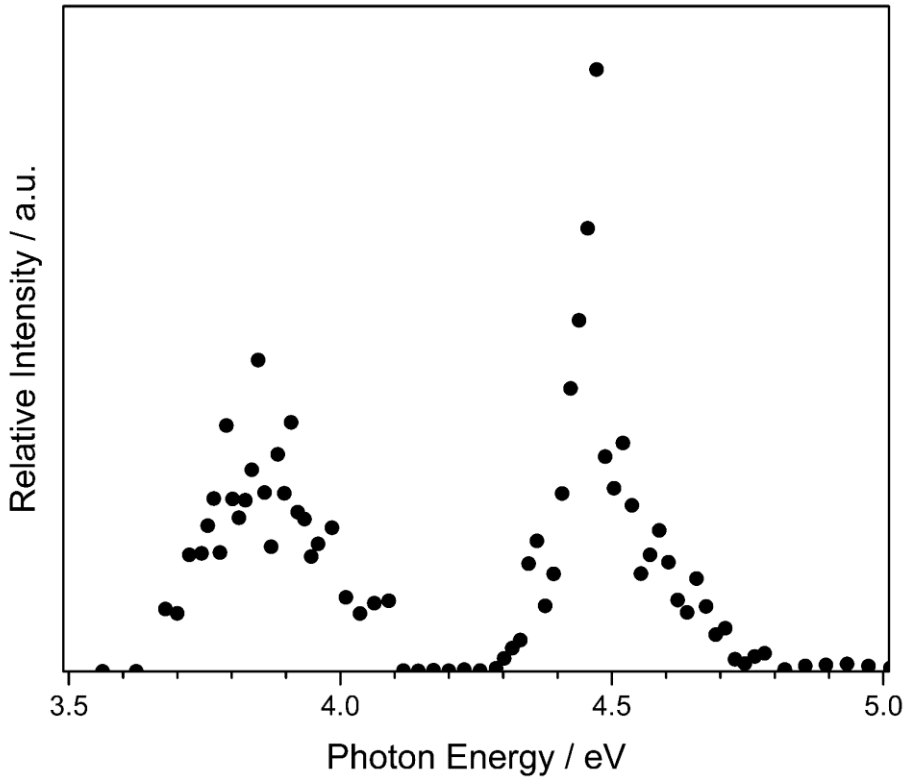

Figure S8: Reconstructed Photodissociation spectrum of Mg^+H_2O for single photon absorption

5. Photon flux dependence

Figures S9 – S14 show the photon flux dependencies of the investigated ions at specific wavelengths.

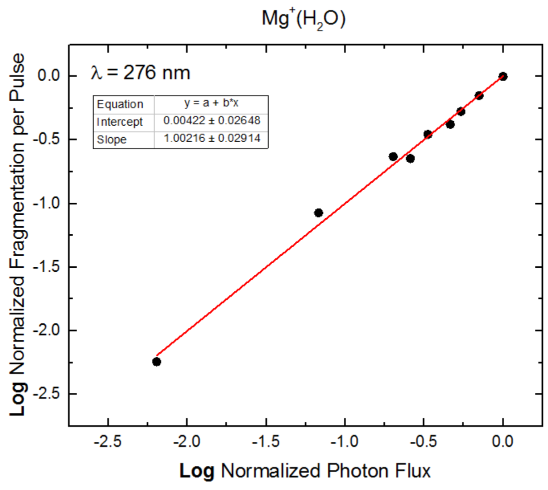

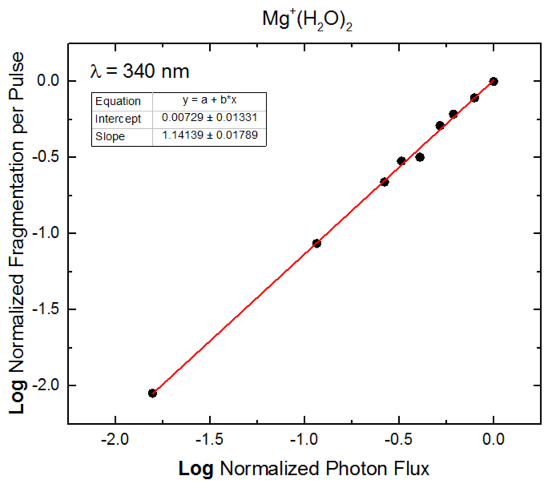

Figure S9: Power dependence of $Mg^{+}(H_2O)$

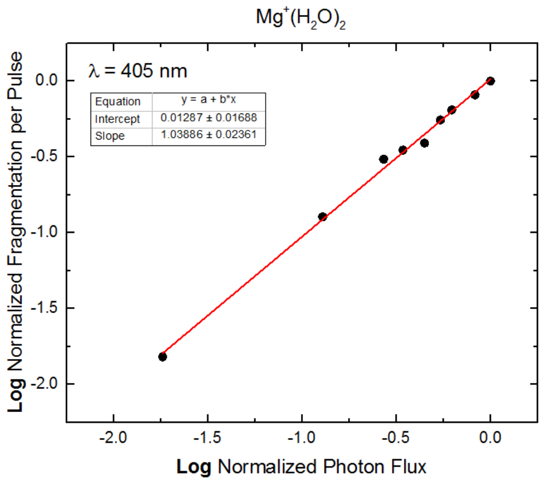

Figure S10: Power dependence of $Mg^{+}(H_2O)_2$

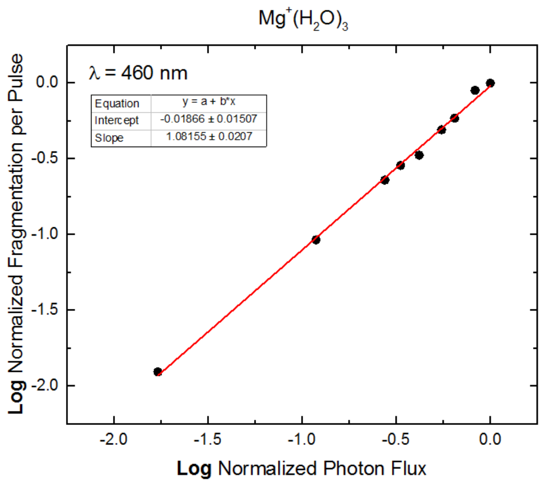

Figure S11 Power dependence of $Mg^{+}(H_2O)_2$

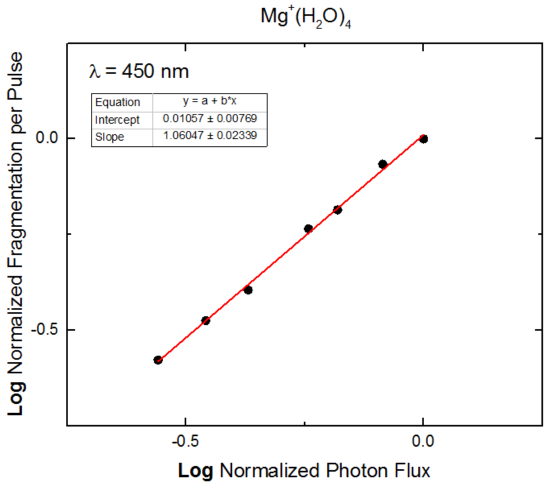

Figure S12: Power dependence of $Mg^{+}(H_2O)_3$

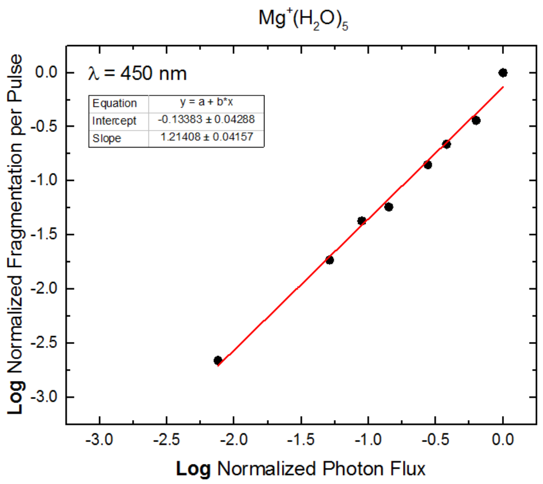

Figure S13: Power dependence of $Mg^{+}(H_2O)_4$

Figure S14: Power dependence of $Mg^{+}(H_2O)_2$

6. Mass spectra

Exemplary mass spectra for the observed ions are shown in Figures S15 – S29 at selected wavelengths, as well as without irradiation.

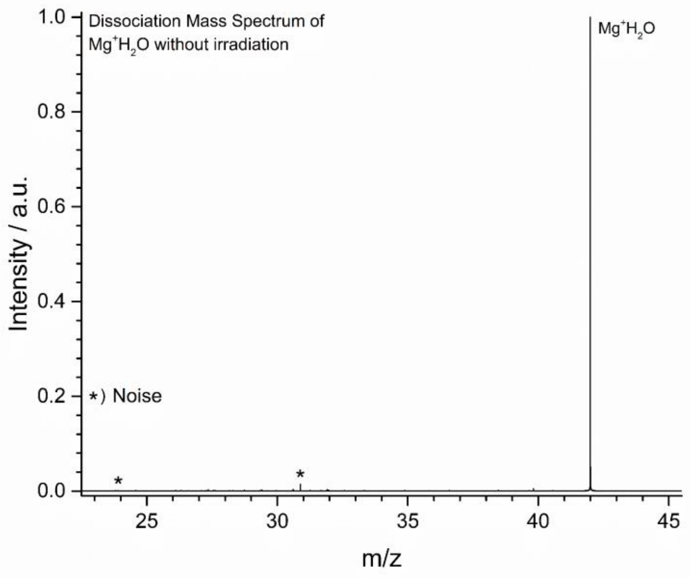

Figure S15: Mass Spectrum of $Mg^{+}H_2O$ without irradiation

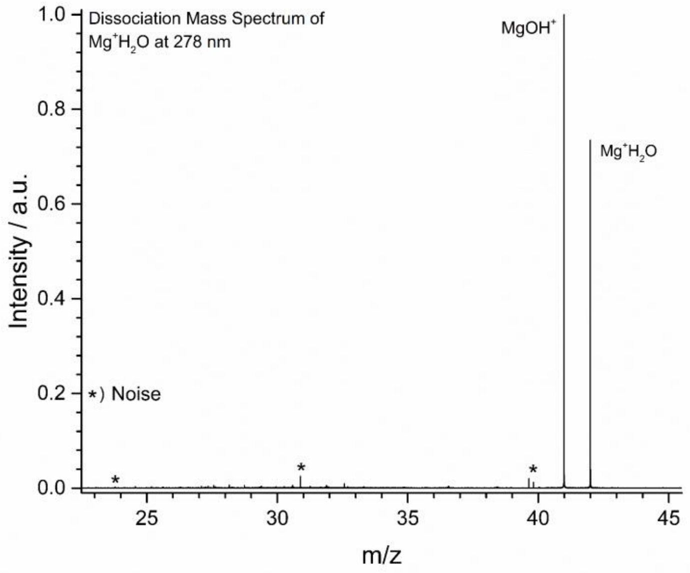

Figure S16: Mass Spectrum of $Mg^{+}H_2O$ after irradiation at 278 nm

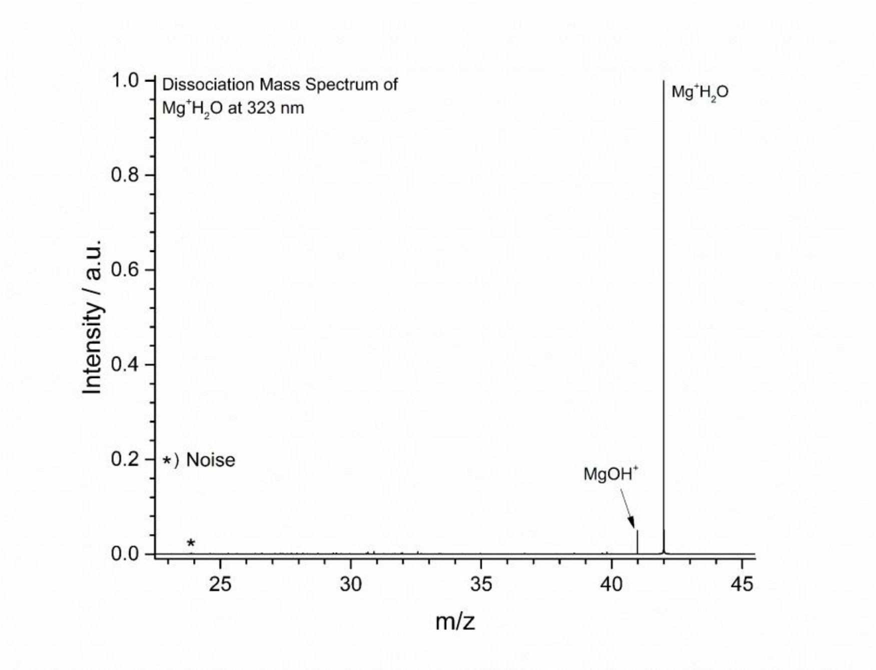

Figure S17: Mass Spectrum of $Mg^{+}H_2O$ after irradiation at 323 nm

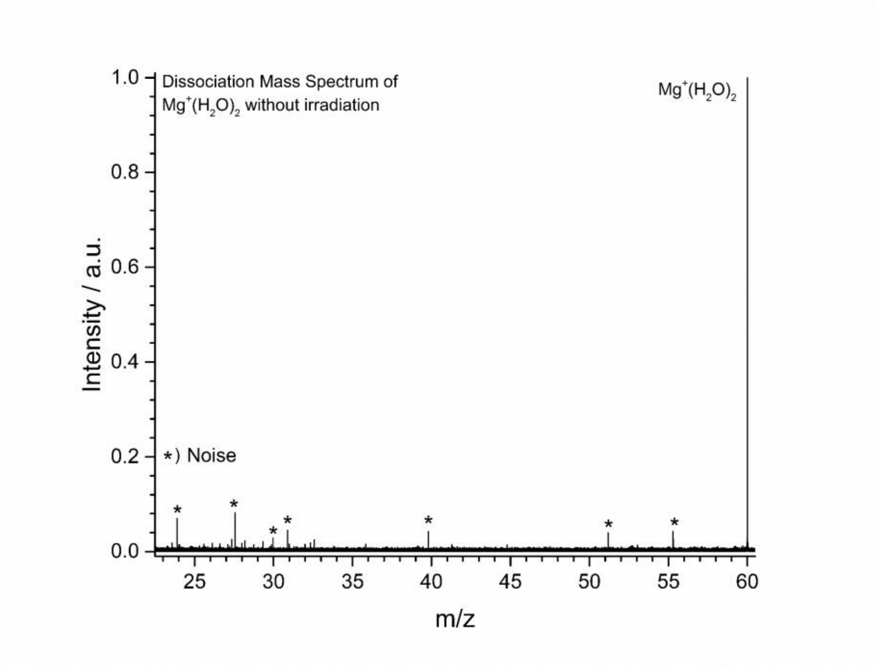

Figure S18: Mass Spectrum of $Mg^{+}(H_2O)_2$ without irradiation

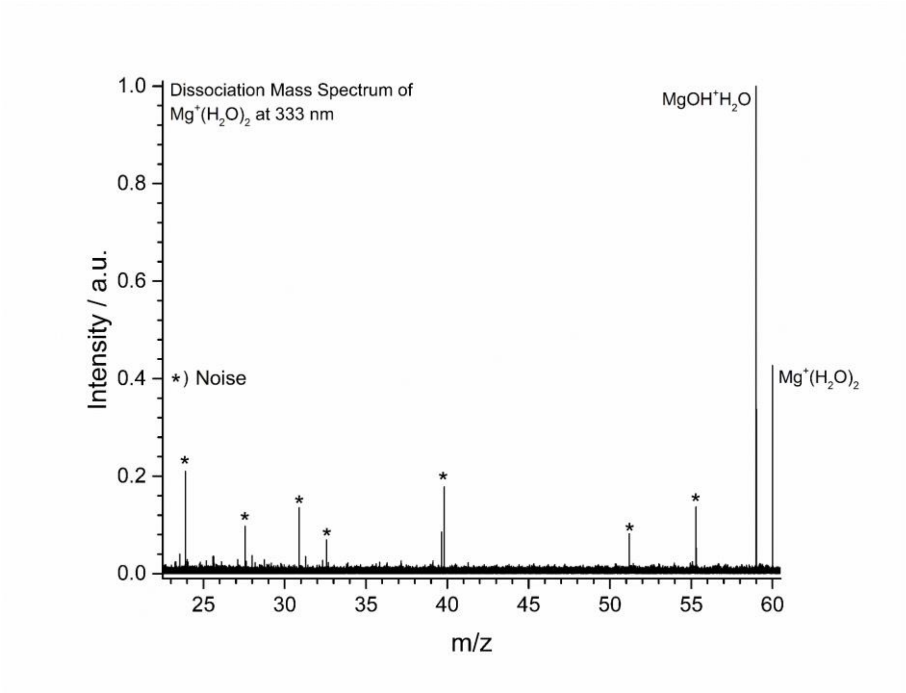

Figure S19: Mass Spectrum of $Mg^{+}(H_2O)_2$ after irradiation at 333 nm

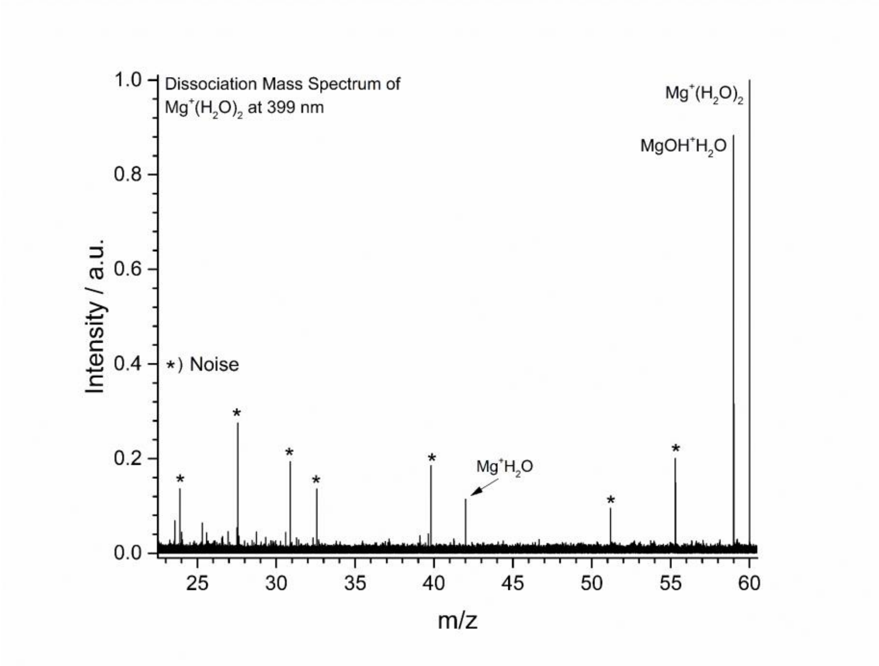

Figure S20: Mass Spectrum of $Mg^{+}(H_2O)_2$ after irradiation at 399 nm

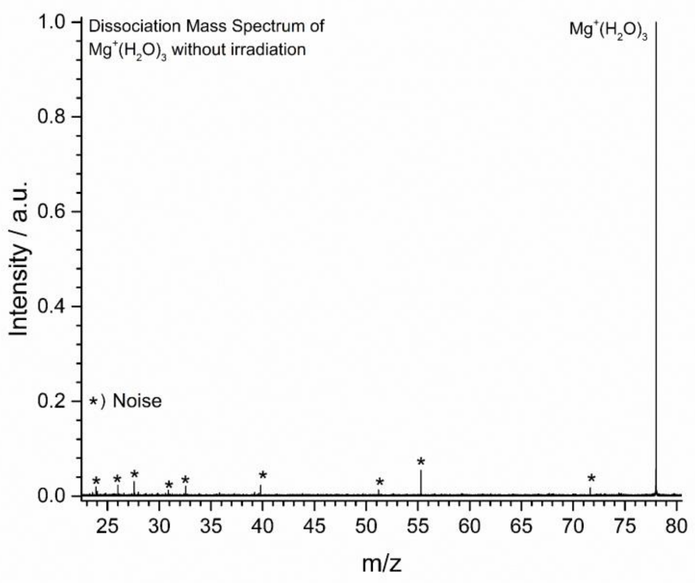

Figure S21: Mass Spectrum of $Mg^{+}(H_2O)_3$ without irradiation

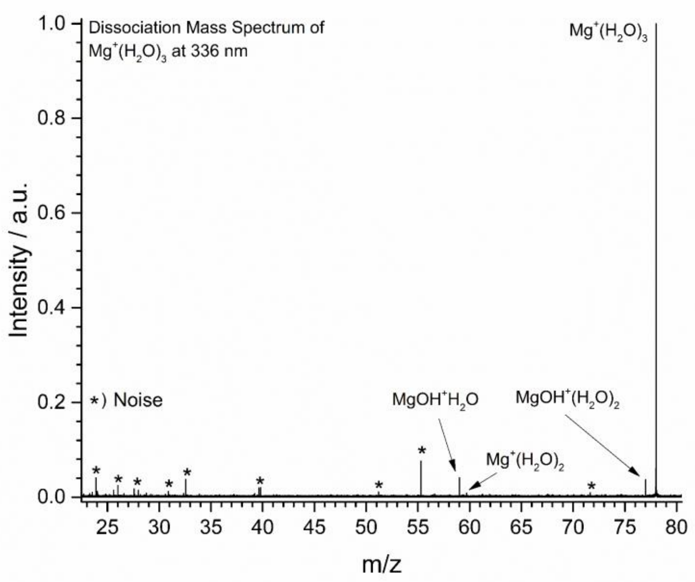

Figure S22: Mass Spectrum of $Mg^{+}(H_2O)_3$ after irradiation at 336 nm

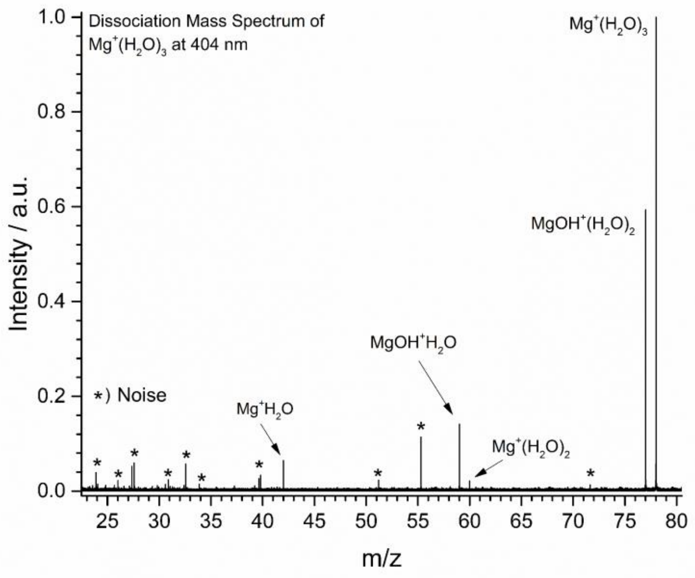

Figure S23: Mass Spectrum of $Mg^+(H_2O)_3$ after irradiation at 404 nm

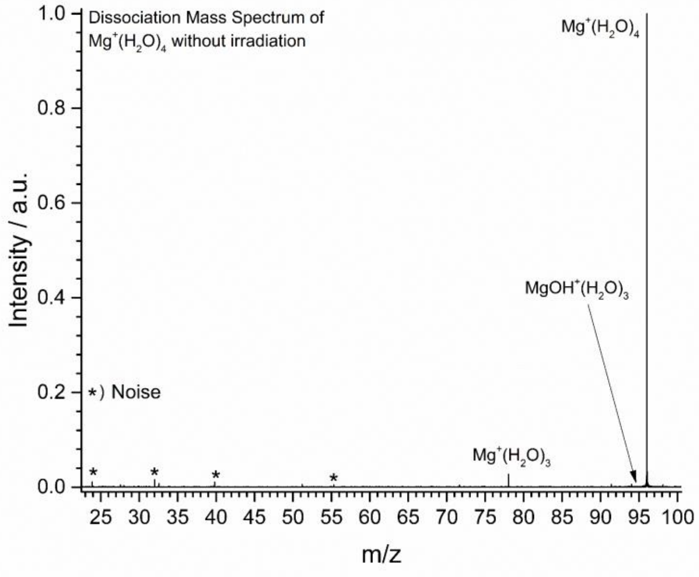

Figure S24: Mass Spectrum of $Mg^+(H_2O)_4$ without irradiation

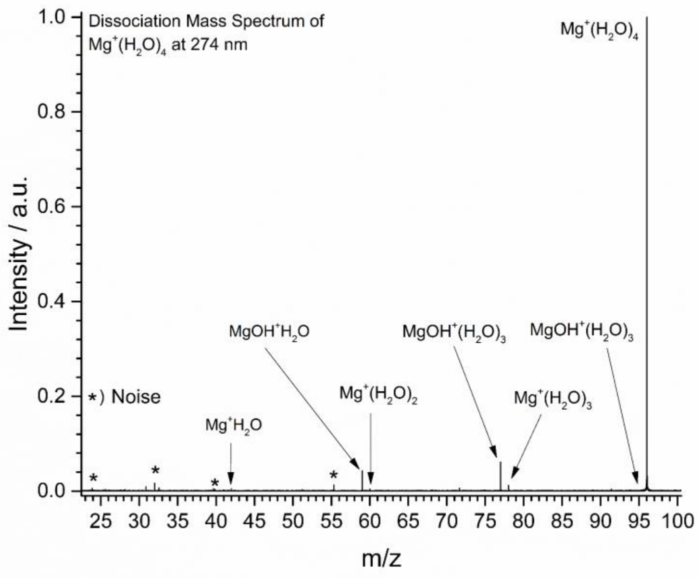

Figure S25: Mass Spectrum of $Mg^+(H_2O)_4$ after irradiation at 274 nm

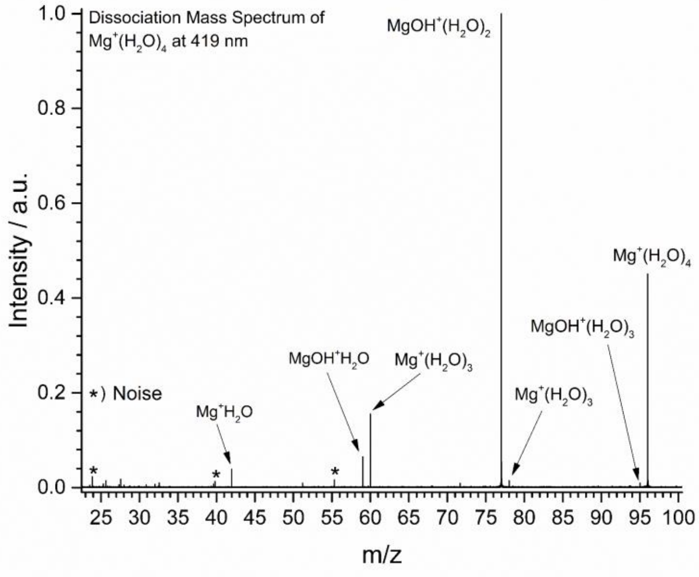

Figure S26: Mass Spectrum of $Mg^+(H_2O)_4$ after irradiation at 419 nm

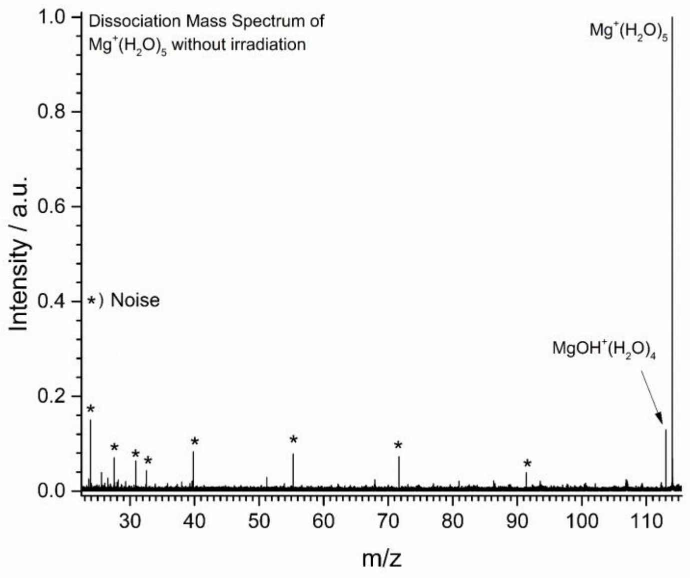

Figure S27: Mass Spectrum of $Mg^{+}(H_2O)_5$ without irradiation

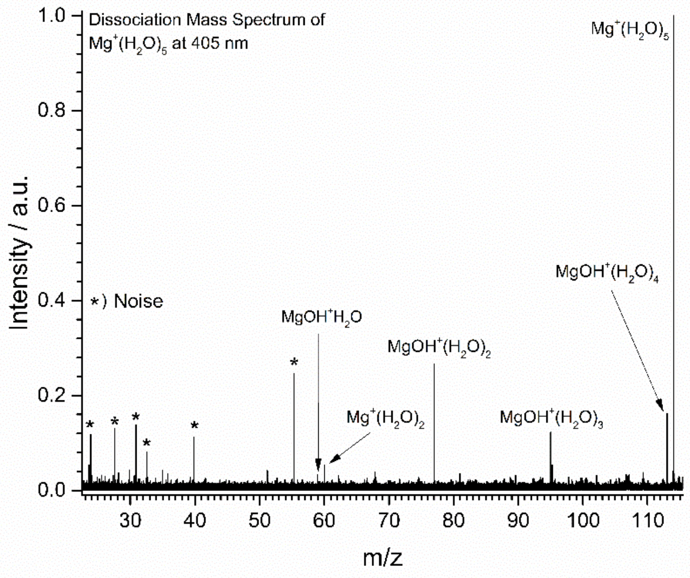

Figure S28: Mass Spectrum of $Mg^{+}(H_2O)_5$ after irradiation at 405 nm

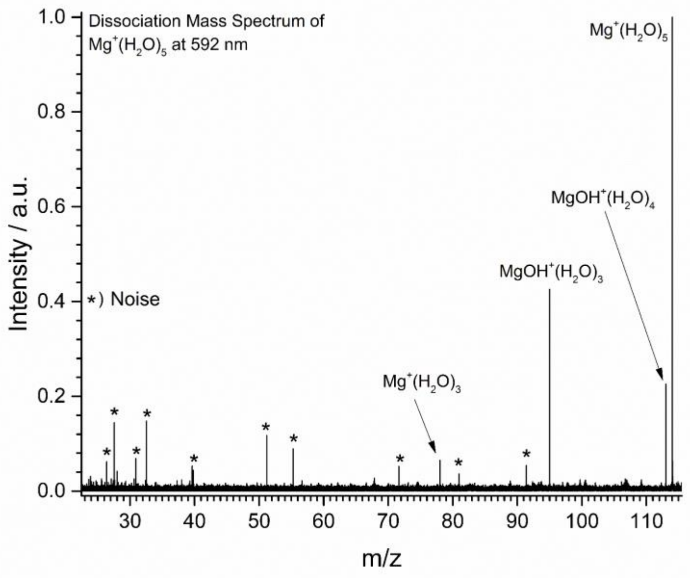

Figure S29: Mass Spectrum of $Mg^+(H_2O)_5$ after irradiation at 592 nm

8. Correction of jumps in the spectra

At the wavelength where the laser system switches from SFG/SH generation to idler beam, jumps appear in the relative photodissociation cross sections, most likely due to a spatial deviation of the two different beams. To correct this, the relative cross sections were adjusted to meet at the same level at this specific wavelength, by multiplying the intensities at lower wavelengths by a constant factor. For Mg^+H_2O no such correction was necessary as the whole spectrum is located in the wavelength range above 410 nm. For $Mg^+(H_2O)_2$ a correction factor of 2.0 was used (Figure S30).

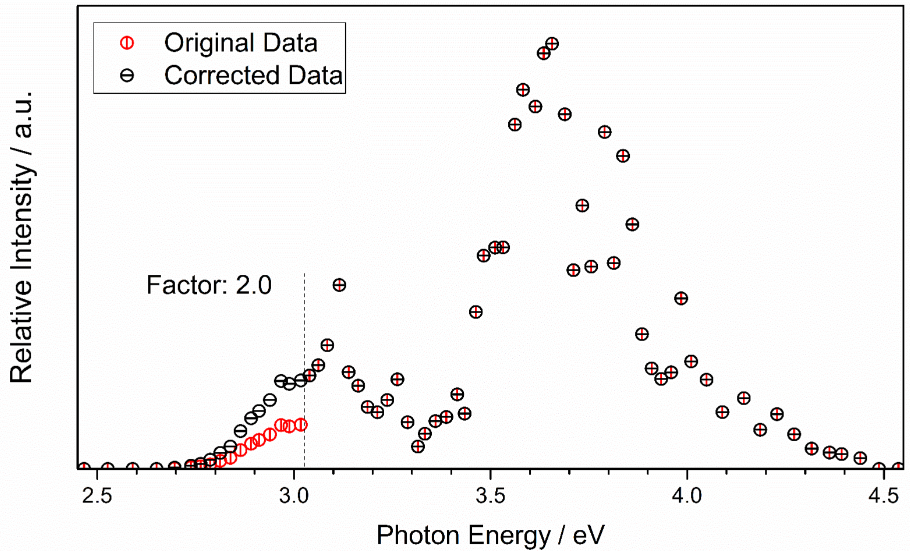

Figure S30: Jump correction in the spectrum of $Mg^+(H_2O)_2$ above 410 nm

For $Mg^+(H_2O)_3$ the applied correction factor was 5.5 (Figure S31).

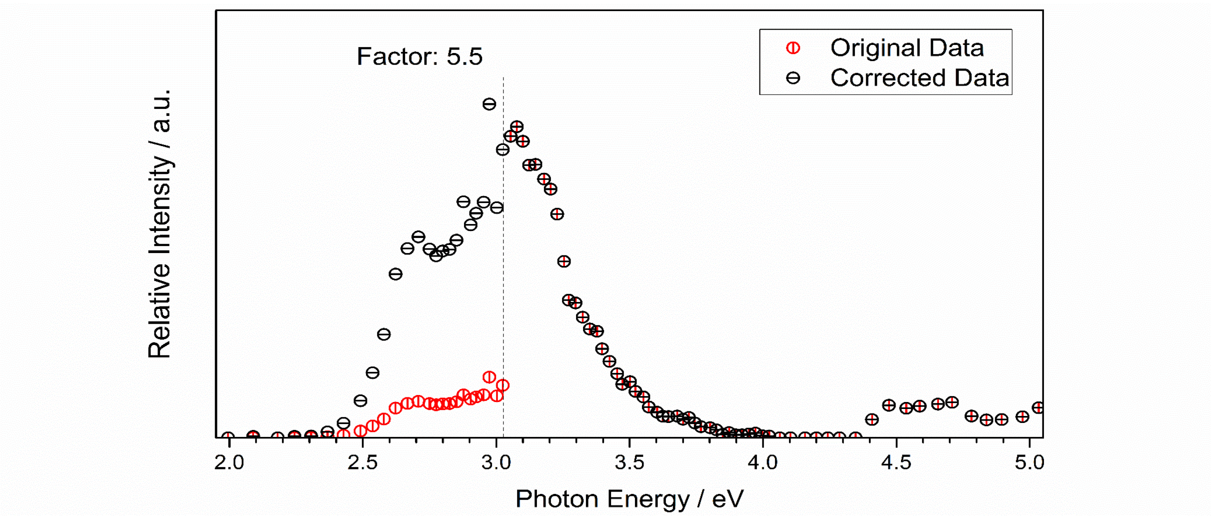

Figure S31: Jump correction in the spectrum of $Mg^{+}(H_2O)_3$ above 410 nm

For $Mg^{+}(H_2O)_4$ the correction factor chosen was 0.9 (Figure S32). The original data here has already been corrected for BIRD (for details see section 9).

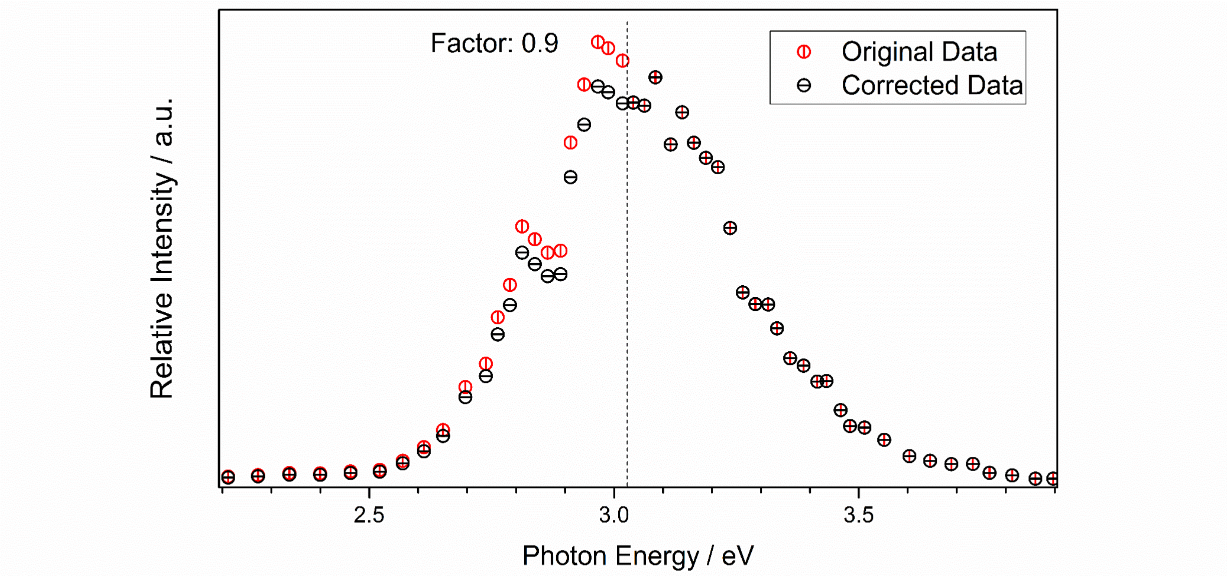

Figure S32: Jump correction in the spectrum of $Mg^{+}(H_2O)_4$ above 410 nm

For $Mg^{+}(H_2O)_5$ the correction factor chosen was 6.0 (Figure S33). The original data here has already been corrected for BIRD (for details see section 9).

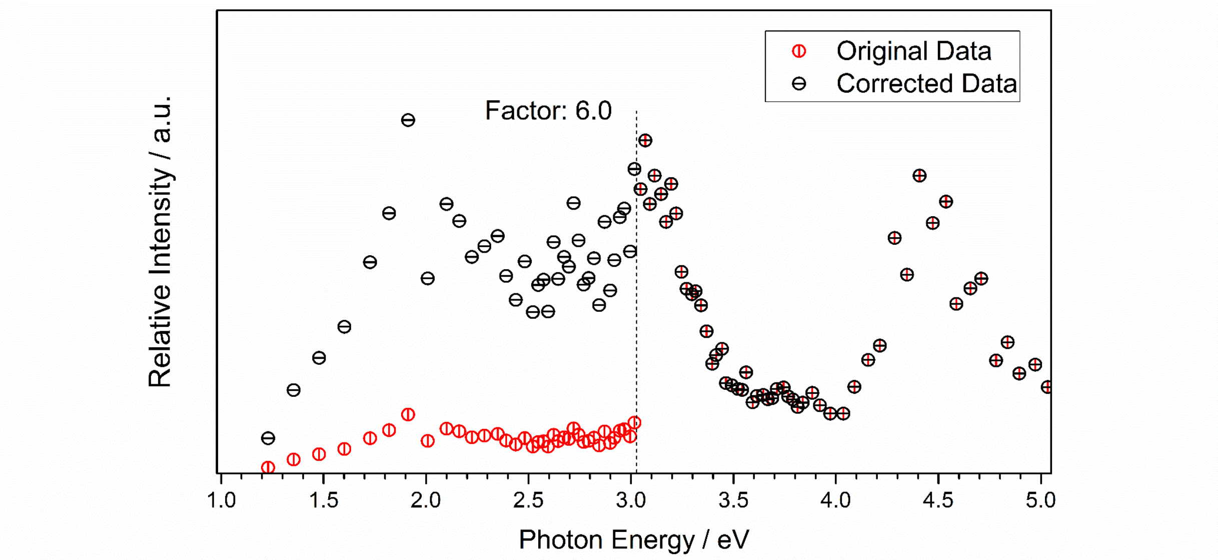

Figure S33: Jump correction in the spectrum of $Mg^+(H_2O)_5$ above 410 nm

9. BIRD corrections

Corrections for BIRD were done according to Equation 1 in section 2, where k_{BIRD} was derived from Equation 2.

Equation 2: $\frac{I_{BIRD-Fragments}}{I_{Photo-Fragments}} = \frac{k_{BIRD}}{\sigma\, \Phi}$ with: $\Phi = \frac{\lambda\, p\, E}{h\, c\, A}/t_{IR}$ and: $t_{IR} = p\, t_p$

Here $I_{BIRD\text{-}Fragments}$ is the intensity of fragments produced by BIRD and $I_{Photo\text{-}Fragments}$ is the intensity of fragments produced by photodissociation. The photon flux is represented by Φ while t_{IR} stands for the irradiation time and t_p for the pulse length.

For $Mg^+(H_2O)_5$ only one BIRD fragment $MgOH^+(H_2O)_4$ was observed and it was completely independent from laser irradiation. Its intensity without laser irradiation was in the order of 10-20% in the timescale of the experiment. The correction is shown in Figure S34.

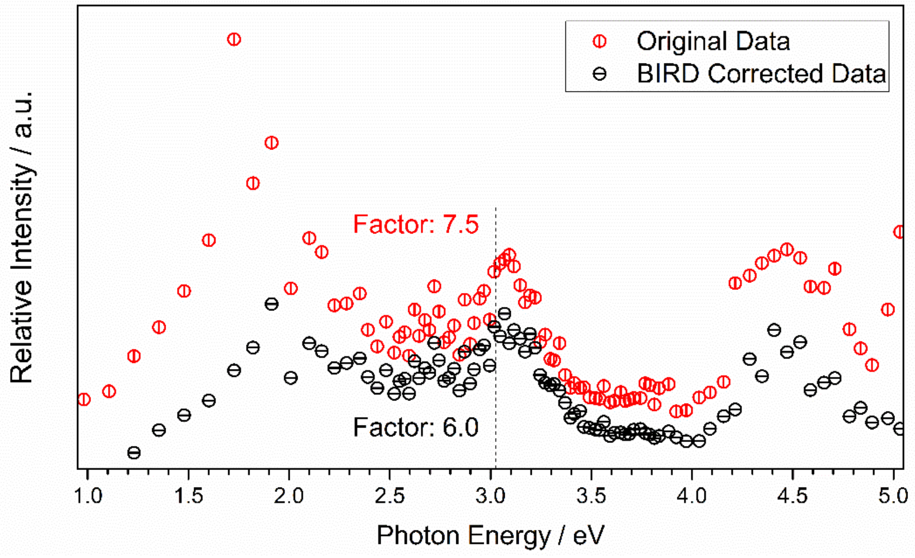

Figure S34: BIRD correction in the spectrum of $Mg^{+}(H_2O)_5$

For $Mg^{+}(H_2O)_4$ two BIRD fragments were observed, $MgOH^{+}(H_2O)_3$ and $Mg^{+}(H_2O)_3$. Only a minor amount of about 4% of the parent ions dissociated via BIRD without laser irradiation on the timescale of the experiment. The intensity of the $Mg^{+}(H_2O)_3$ fragment was thereby also completely independent form laser irradiation, while $MgOH^{+}(H_2O)_3$ shows wavelength dependency in the range from 393 nm to 688 nm. In this region the ratio of the $MgOH^{+}(H_2O)_3$ fragment created by BIRD was estimated to be 0.1 % of the $Mg^{+}(H_2O)_3$ fragment. The correction is shown in Figure S35.

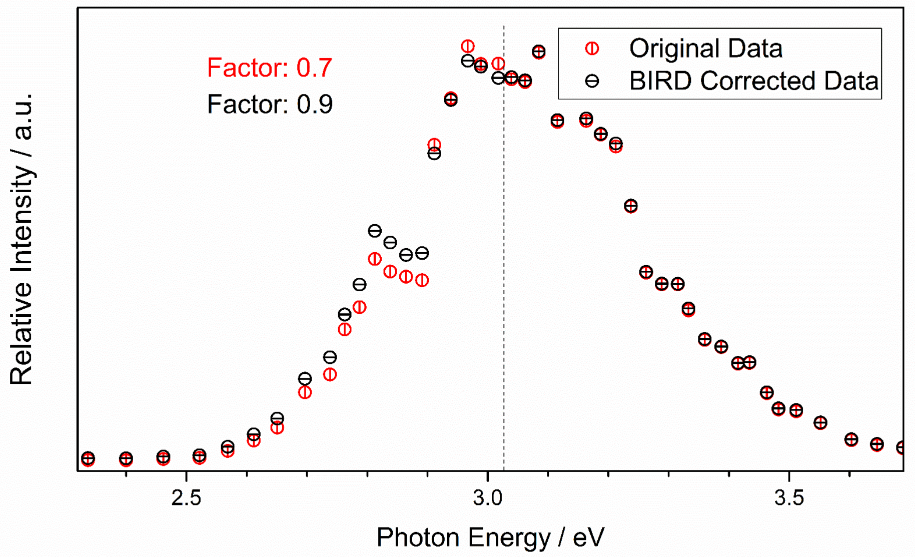

Figure S35: BIRD correction in the spectrum of $Mg^{+}(H_2O)_4$

10. Further benchmarks of computational methods

Figure S36 shows the photoabsorption spectra of the $Mg^+(H_2O)_2$ cluster using a different number of random walkers within the PIMD sampling. It can be used that there are only limited changes between spectra produced from PIMD simulations with 10 and 20 random walkers, the most significant difference is the height of the peak at 3.75 eV. Otherwise, the shape of the spectrum is almost unaffected.

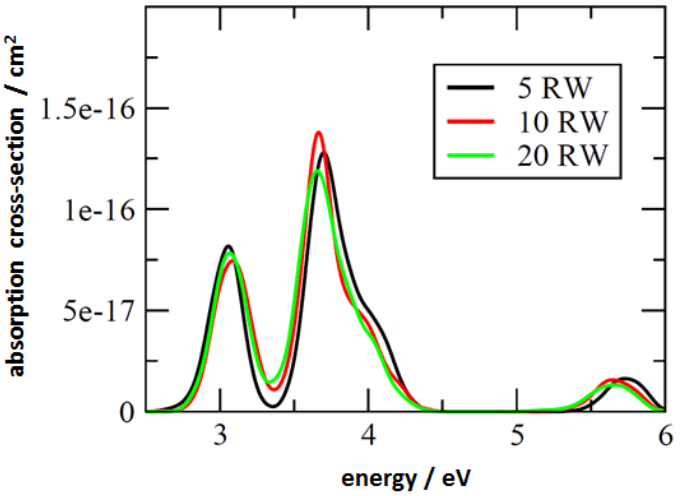

Figure S36: Photoabsorption spectra of the $Mg^+(H_2O)_2$ cluster calculated at the CC2/aug-cc-pVDZ level of theory with a different number of random walkers used within the PIMD sampling.

Figure S37 compares relaxed potential at the EOM-CCSD and MRCI method. MRCI can quantitatively reproduce energy of first four states in doublet multiplicity, D_0 and D_1-D_3. For the fourth excited state D_4, EOM-CCSD and MRCI differ already slightly, the agreement is however still very good. Finally, the fifth excited state D_5 state was included into the MRCI state average for completeness to avoid convergence of D_4 into a wrong state. At the MRCI level, the D_5 state is sometimes predicted to be too high in energy (e.g. for the O-H coordinate at 1.2 Å). This is happens when a higher state comes from the CASSCF calculations into the MRCI procedure.

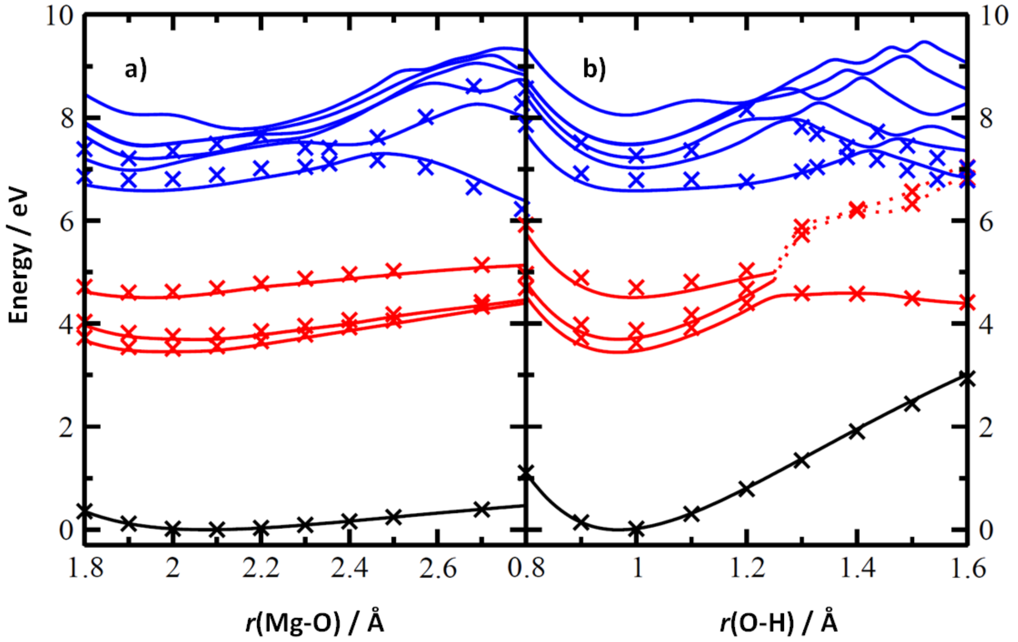

Figure S37: Relaxed potential energy scans for Mg^+H_2O along a) Mg-O and b) O-H dissociation coordinate. Calculated at the EOM-CCSD/aug-cc-pVDZ level of theory (lines) and single-point recalculated at the MRCI(5,9)/aug-cc-pVDZ level with 6 explicitly included states (crosses). The respective curves were optimized in the D_0 (black), D_1 (red) and D_4 (blue) states. Dotted lines are used for clarity when different bands cross.

Structure of calculated clusters as optimized at the MP2/def2TZVP level of theory (Cartesian coordinates, in Å) along with the respective electronic energy (including zero-point energy correction, in Hartree)

H
E=-0.499810

H 0. 0. 0.

H2
E=-1.149944

H 0.000000 0.015969 0.000000
H 0.000000 0.753413 0.000000

OH
E=-75.609006

O 0.000000 0.000479 0.693118
H 0.000000 0.781542 1.271580

H2O
E=-76.291690

O 0.000000 0.000000 0.686599
H 0.000000 0.761339 1.275017
H 0.000000 -0.761339 1.275017

Mg+
E=-199.363680

Mg 0.000000 0.000000 -1.396538

Mg(H2O)+
E=-275.705708

Mg 0.000000 0.000000 -1.378467
O 0.000000 0.000000 0.688243
H 0.000000 0.775776 1.270965
H 0.000000 -0.775776 1.270965

Mg(H2O)2+, iso1
E=-352.038053

mg 0.014827 0.000000 0.015029
o 0.002909 0.001308 2.108193
o 2.107963 -0.001308 0.031313
h -0.745641 -0.313592 2.637300
h 0.551117 0.532978 2.704325
h 2.647107 0.313592 -0.710040
h 2.696654 -0.532978 0.587504

Mg(H2O)2+, iso2
E=-352.027410

o 0.000000 0.000000 0.000000
mg 0.000000 0.000000 2.001014
o 2.210474 0.000000 -1.319852
h 0.827925 0.000000 -0.575021
h -0.765552 0.000000 -0.590976
h 2.603556 -0.772876 -1.744744
h 2.603556 0.772876 -1.744744

Mg(H2O)3+, iso1
E=-428.364230

Mg 0.000000 0.000000 0.000000
O 1.710894 0.000000 1.254897
O -0.855447 -1.481678 1.254897
O -0.855447 1.481678 1.254897
H 2.583467 0.290192 0.951813
H 1.868472 -0.679276 1.926618
H -1.040420 -2.382445 0.951813
H -1.522506 -1.278507 1.926618
H -1.543048 2.092252 0.951813
H -0.345966 1.957783 1.926618

Mg(H2O)3+, iso2
E=-428.359488

mg 0.000000 0.000000 0.010984
o 0.000000 -1.409167 1.520530
o 0.000000 1.409167 1.520530
h 0.000000 -1.184047 2.473162
h 0.000000 -2.371196 1.431239
h 0.000000 1.184047 2.473162
h 0.000000 2.371196 1.431239
o 0.000000 0.000000 3.904147
h 0.768723 0.000000 4.493216
h -0.768723 0.000000 4.493216

Mg(H2O)3+, iso3
E=-428.343785

mg 0.000000 0.000000 0.000000
o 0.000000 0.000000 1.964103
h 0.811135 0.000000 2.538419
h -0.811135 0.000000 2.538419
o 2.276091 0.000000 3.283563
o -2.276091 0.000000 3.283563
h 2.693183 0.770776 3.687427
h 2.693183 -0.770776 3.687427
h -2.693183 0.770776 3.687427
h -2.693183 -0.770776 3.687427

Mg(H2O)4+, iso1
E=-504.679325

mg 0.042174 0.000000 0.165309
o 0.035965 0.000000 2.293156
o 2.155645 0.000000 0.227076
o 0.363995 2.160882 0.169931
o 0.363995 -2.160882 0.169931
h 0.814882 0.000000 2.866068
h -0.741855 0.000000 2.867059
h 2.659276 -0.778175 -0.048163
h 2.659276 0.778175 -0.048163
h -0.084075 2.599083 -0.569954
h 0.258020 2.752661 0.929301
h 0.258020 -2.752661 0.929301
h -0.084075 -2.599083 -0.569954

Mg(H2O)4+, iso2
E=-504.681639

Mg 0.225253 0.000000 0.032859
O 1.955983 0.000000 1.222827
O -0.820890 -1.410416 1.183832
O -0.820890 1.410416 1.183832
H 2.848178 0.000000 0.849740
H 2.048042 0.000000 2.185185
H -1.649954 -1.183991 1.648383
H -0.834160 -2.358382 0.999137
H -1.649954 1.183991 1.648383
H -0.834160 2.358382 0.999137
O -2.928553 0.000000 2.353895
H -3.080548 0.000000 3.309689
H -3.812150 0.000000 1.958558

Mg(H2O)4+, iso3
E=-504.673302

mg 0.000419 -0.000808 -0.000330
o 1.395235 0.004598 1.505984
o -1.394958 0.003964 1.505466
o -0.000283 -0.008282 3.800711
o -0.000004 -2.203806 5.311253
h 2.358319 0.014321 1.446474
h 1.128026 0.001452 2.455563
h -1.128099 0.000945 2.455143
h -2.358024 0.013278 1.445603
h -0.000183 -0.808355 4.387296
h -0.000530 0.754756 4.394300
h 0.769575 -2.613091 5.724113
h -0.769493 -2.613503 5.723869

Mg(H2O)4+, iso4
E=-504.652556

Mg -0.313799 -0.107312 -1.038017
O 0.209105 -0.001342 0.838287
H 0.249856 0.817830 1.411135
H 0.260190 -0.752610 1.496957
O 0.143101 1.933283 2.578944
O 0.167120 -1.735489 2.779413
H 0.642609 2.750355 2.685486
H 0.677158 -2.529506 2.973998
H 0.016140 1.554739 3.466051
H 0.034589 -1.264234 3.620085
O -0.324523 0.214074 4.844488
H 0.227479 0.260999 5.637139
H -1.233092 0.226257 5.176258

Mg(H2O)5+, iso1
E=-580.992031

O 0.000000 0.000000 -0.000928
Mg 0.000000 0.000000 2.134528
O -2.139164 0.060050 2.054370
O 2.139164 -0.060050 2.054370
O 0.060790 -2.136293 1.751477
O -0.060790 2.136293 1.751477
H 0.001810 -0.781769 -0.566691
H -0.001810 0.781769 -0.566691
H -2.573919 -0.009414 2.918563
H 2.573919 0.009414 2.918563
H -2.788666 -0.187179 1.379266
H 2.788666 0.187179 1.379266
H 0.583065 2.659708 2.251840
H 0.921424 -2.523265 1.973404
H -0.921424 2.523265 1.973404
H -0.583065 -2.659708 2.251840

Mg(H2O)5+, iso2
E=-580.994606

O -0.151982 -1.995744 0.754222
Mg -0.134507 -0.564653 -0.818808
O -2.182265 -0.247165 -0.137526
O 2.037394 -0.699456 -0.730993
O 0.201059 1.199455 0.262608
H -0.972258 -2.065826 1.262138
H 0.192685 -2.894079 0.664254
H 1.044744 1.664136 0.207966
H -0.494222 1.850772 0.472542
H 2.636325 -1.006221 -0.035253
H 2.470936 -0.883143 -1.577170
H -2.469760 0.645212 0.135424
H -2.851176 -0.575333 -0.755184
O -2.247963 2.461524 0.696484
H -2.553736 2.716408 1.578106
H -2.569683 3.157160 0.106030

Mg(H2O)5+, iso3
E=-580.994740

O 0.548506 -1.212800 0.144386
Mg 1.349633 0.035817 -1.279103
O -0.411967 -0.285075 -2.367010
O 2.522889 -1.480477 -2.145253
O -2.214488 -1.442290 -0.611209
H -0.401668 -1.415272 0.166697
H 0.892344 -1.264149 1.069848
H -1.216782 -0.642677 -1.942152
H -0.680595 0.221371 -3.143382
H -2.895062 -0.917765 -0.166916
H -2.630854 -2.297075 -0.788259
H 3.381339 -1.325334 -2.561070
H 2.384740 -2.436373 -2.113973
O 1.479815 -1.278570 2.642678
H 1.683470 -0.487152 3.155000
H 1.909471 -2.010399 3.100306

Mg(H2O)5+, iso4
E=-580.981452

O 0.385702 0.638850 0.581817
Mg -0.890873 0.794069 -0.977371
O 0.542118 2.146687 2.722567
O -0.383986 -1.648323 1.975539
O -0.637331 -0.058575 4.329263
H 0.557593 1.316256 1.287058
H 0.448462 -0.220341 1.039151
H 1.116235 2.852322 3.040451
H 0.262557 1.627458 3.491140
H -0.006432 -2.521103 2.144868
H -0.553446 -1.244797 2.850571
H -0.177377 -0.366373 5.122801
H -1.527578 0.164040 4.635751
O -1.725018 -0.984314 -0.312270

H -2.418737 -1.524082 -0.710462
H -1.443576 -1.397214 0.533306

Mg(H2O)5+, iso5
E=-580.962173

mg 0.018788 0.000000 0.069137
o -0.029694 0.000000 2.012187
o 0.552397 -1.818096 3.757347
o 0.552397 1.818096 3.757347
o 1.626082 0.000000 5.591394
o 0.769430 0.000000 8.172300
h 0.100723 -0.787567 2.621377
h 0.100723 0.787567 2.621377
h 0.052798 -2.565141 4.103902
h 0.052798 2.565141 4.103902
h 0.934738 -1.342572 4.522258
h 0.934738 1.342572 4.522258
h 1.333290 0.000000 6.531774
h 2.591332 0.000000 5.627650
h 0.702896 -0.768036 8.751106
h 0.702896 0.768036 8.751106

Mg(H2O)6+
E=-657.306428

O 1.500762 0.498053 -1.352726
Mg -0.046149 0.016704 0.000158
O -1.526858 -0.818879 1.217732
O -0.500161 1.992821 0.511796
O 0.594099 -1.986554 -0.176479
O 1.398873 0.212566 1.526120
O -1.225336 0.013995 -1.726869
H 1.852220 -0.498681 1.997601
H 1.324036 0.965062 2.130320
H -2.001379 0.604947 -1.576734
H -1.601351 -0.811103 -2.087611
H -0.010758 -2.685583 0.110574
H 1.171722 -2.358581 -0.855911
H 1.254371 0.782481 -2.244521
H 2.358279 0.892153 -1.145243
H -1.468982 2.055643 0.689122
H -0.304429 2.734614 -0.091323
H -2.407435 -0.512561 0.894014
H -1.543533 -0.664858 2.181222

MgOH+
E=-275.100552

Mg 0.000000 -0.139708 -0.733254
O 0.000000 0.309885 0.916378
H 0.000000 0.560958 1.837539

MgOH(H2O)+
E=-351.472484

O -0.041541 0.197447 -1.653069
Mg -0.008853 0.246596 0.071066
O 0.029530 0.304089 2.061046
H -0.060659 0.170061 -2.605720
H -0.229521 -0.405994 2.670095
H 0.311046 1.047949 2.617545

MgOH(H2O)2+
E=-427.825500

O -0.532738 -1.891279 0.672807
Mg -0.804356 0.007476 0.072852
O 0.562141 1.311749 0.757267
O -2.117882 0.508556 -0.961040
H -1.172897 -2.552733 0.366689
H 0.111367 -2.358620 1.221491
H -2.840998 0.762938 -1.525853
H 1.345040 1.269780 1.322461
H 0.460615 2.237459 0.485393

MgOH(H2O)3+
E=-504.165365

O -0.879279 0.763814 1.921530
Mg 0.665434 0.094726 2.557008
O 0.251073 -1.653966 1.617253
O 2.271553 1.179300 1.942529
O 1.278246 -0.572788 4.388701
H -0.589573 -1.374129 1.208248
H -1.587866 1.402031 1.966092
H 0.607618 -2.399465 1.121789
H 0.983021 -1.422622 4.744993
H 2.076792 1.920223 1.349952
H 3.231779 1.093703 1.990796
H 1.546956 -0.037678 5.147885

MgOH(H2O)4+
E=-580.490934

O -0.371406 -1.682467 0.858846
Mg -0.174117 -0.624436 -0.642042
O -1.844276 0.406535 -1.325781
O 0.451199 0.666665 0.935235
O -0.290200 -2.051867 -2.183150
O 1.464042 0.183522 -1.589149
H -0.516033 -2.536155 1.262633
H 1.993496 -0.191223 -2.303776
H 2.054771 0.703443 -1.026362
H -0.296402 -2.963498 -1.859528
H -0.799915 -2.046200 -3.002787
H 0.149713 1.446498 1.413173
H 0.228736 -0.139728 1.476164
H -2.700781 0.231077 -0.912032
H -1.888884 1.303148 -1.681479

MgH+
E=-199.920646

Mg 0.000000 0.000000 -0.366761
H 0.000000 0.000000 1.287122

MgH(H2O)+
E=-276.284915

O 0.000000 -1.373731 0.422330
Mg 0.000000 0.534896 -0.221680
H 0.000000 2.101931 -0.750772
H 0.000000 -1.679294 1.343431
H 0.000000 -2.174679 -0.125650

MgH(H2O)2+
E=-352.631887

O 0.000000 1.732944 0.658587
Mg 0.000000 0.203601 -0.686411
O 0.000000 -1.519749 0.398786
H 0.000000 1.787792 1.624146
H 0.000000 2.646880 0.333672
H 0.000000 -1.727608 1.343293
H 0.000000 -2.370275 -0.067538
H 0.000000 0.336071 -2.349346

MgH(H2O)3+
E=-428.967895

O -0.497306 -1.655646 0.534157
Mg 0.132796 -0.088615 -0.668747
O 2.132617 -0.611480 -0.833741
O 0.316503 1.368819 0.794736
H -0.626805 -1.669836 1.491780
H -1.097707 -2.320930 0.167262
H 1.112094 1.666568 1.255607
H -0.296199 2.118674 0.794875
H 2.598606 -1.356495 -0.431465
H 2.611432 -0.396966 -1.647632
H -0.737277 0.265296 -2.070292

MgH(H2O)4+
E=-505.288707

O -0.311270 -2.049672 0.752116
Mg -0.129945 -0.541155 -0.694020
O -2.214399 -0.004207 -0.621320
O 2.023563 -0.566801 -0.676903
O 0.110982 1.157489 0.482739
H -0.293043 -2.008189 1.716071
H -0.437592 -2.977664 0.513316
H 0.957645 1.619817 0.537461
H -0.584083 1.824456 0.557708
H 2.707355 -0.985028 -0.138757
H 2.274983 -0.709146 -1.602372
H -2.518023 -0.072834 -1.539508
H -2.969490 -0.231493 -0.064304
H -0.195576 -0.868871 -2.382653

MgO+
E=-274.397300

Mg 0.000000 0.000000 -0.452712
O 0.000000 0.000000 1.373073

MgO(H2O)+
E=-350.770390

O 0.000000 -1.399600 0.431057
Mg 0.000000 0.488208 -0.205810
O 0.000000 2.226398 -0.792861
H 0.000000 -1.704945 1.353114
H 0.000000 -2.200938 -0.117839

MgO(H2O)2+
E=-427.125542

O 1.696174 -0.886155 0.000165
Mg -0.000049 0.194139 0.000182
O -0.001901 2.043108 -0.000085
O -1.694666 -0.888598 -0.000230
H 2.538051 -0.429674 -0.158459
H 1.914748 -1.805367 0.208137
H -2.537336 -0.433282 0.157529
H -1.911724 -1.808175 -0.208185

MgO(H2O)3+
E=-503.468395

O -0.517339 -1.756557 0.575820
Mg 0.240760 -0.161231 -0.436618
O 0.453802 1.573745 0.607182
O 2.174895 -0.720191 -0.784984
H -0.378464 -2.224799 1.408572
H -1.352866 -2.074955 0.199889
H 0.821053 1.880614 1.445608
H -0.079035 2.297176 0.241752
H 2.448299 -1.603108 -1.071140
H 2.880351 -0.116925 -1.058060
O -1.042701 0.225620 -1.741479

MgO(H2O)4+
E=-579.795412

O -0.311576 -2.061003 0.840203
Mg -0.126776 -0.532816 -0.535978
O 0.117599 1.200185 0.555691
O -2.166677 0.047079 -0.695954
O 1.988958 -0.504540 -0.750418
H -0.308021 -2.129238 1.802501
H -0.434735 -2.953956 0.488195
H 0.964248 1.660740 0.624579
H -0.577487 1.865456 0.644796
H 2.698675 -0.959913 -0.280518
H 2.164259 -0.635421 -1.695859
H -2.394413 -0.030368 -1.636089
H -2.958245 -0.208905 -0.206300
O -0.234700 -1.170596 -2.315276
